# NAD^+^ augmentation with nicotinamide riboside improves lymphoid potential of *Atm*^−/−^ and old mice HSCs

**DOI:** 10.1038/s41514-021-00078-3

**Published:** 2021-09-21

**Authors:** Le Zong, Mayuri Tanaka-Yano, Bongsoo Park, Hagai Yanai, Ferda T. Turhan, Deborah L. Croteau, Jane Tian, Evandro F. Fang, Vilhelm A. Bohr, Isabel Beerman

**Affiliations:** 1 Epigenetics and Stem Cell Unit, Translational Gerontology Branch, National Institute on Aging, NIH, Baltimore, MD 21224 USA; 2 Laboratory of Molecular Gerontology, National Institute on Aging, NIH, Baltimore, MD 21224 USA; 3 Department of Clinical Molecular Biology, University of Oslo and Akershus University Hospital, 1478 Lørenskog, Norway

**Keywords:** Ageing, Adult stem cells

## Abstract

NAD^+^ supplementation has significant benefits in compromised settings, acting largely through improved mitochondrial function and DNA repair. Elevating NAD^+^ to physiological levels has been shown to improve the function of some adult stem cells, with implications that these changes will lead to sustained improvement of the tissue or system. Here, we examined the effect of elevating NAD^+^ levels in models with reduced hematopoietic stem cell (HSC) potential, ATM-deficient and aged WT mice, and showed that supplementation of nicotinamide riboside (NR), a NAD^+^ precursor, improved lymphoid lineage potential during supplementation. In aged mice, this improved lymphoid potential was maintained in competitive transplants and was associated with transcriptional repression of myeloid gene signatures in stem and lineage-committed progenitor cells after NR treatment. However, the altered transcriptional priming of the stem cells toward lymphoid lineages was not sustained in the aged mice after NR removal. These data characterize significant alterations to the lineage potential of functionally compromised HSCs after short-term exposure to NR treatment.

## Introduction

Nicotinamide adenine dinucleotide (oxidized form, NAD^+^) is a critical coenzyme with broad physiological functions, including the regulation of metabolism and DNA repair. Decreased levels of NAD^+^ are often associated with impaired conditions, such as those found in models of cancer, metabolic disorders, neurodegeneration, as well as physiological and accelerated aging processes^1–4^. Recent studies have highlighted significant improvements of compromised systems after NAD^+^ levels have been elevated through supplementation with NAD^+^ precursors including nicotinic acid, nicotinamide, nicotinamide mononucleotide, or nicotinamide riboside (NR)^5–8^.

We recently examined the role of NAD^+^ supplementation in models of ataxia–telangiectasia (A–T), which is a disorder caused by mutations (ATM)^9–11^. A–T is a rare autosomal recessive disease with patients showing uncoordinated movement, telangiectasia, radiosensitivity, severe cerebellar atrophy, and distinct features of accelerated aging^12^. Murine models of ATM loss show defects in DNA damage repair associated with mitochondrial dysfunction^13^ and loss of hematopoietic stem cell (HSC) potential^14^. Supplementation of NR in models of A–T led to significantly improved lifespan and healthspan, mediated by improvement of both DNA damage repair and mitophagy in the tissues examined^1,11,15^. However, the role of NR supplementation on the hematopoietic system and stem cell compartment has not yet been explored in this model.

The hematopoietic system includes the cells that give rise to and reside in the blood. Blood cells can be divided into three lineages: myeloid, lymphoid, and erythroid. Myeloid cells have roles in innate immunity and include neutrophils, megakaryocytes, and macrophages. Lymphoid cells, including B and T cells, are responsible for adaptive immunity. Erythroid cells, or red blood cells, are key for the oxygen transport to cells throughout the body and deliver carbon dioxide to the lungs. Alterations in the balance of these cell types can lead to compromised function of the blood system. HSCs are responsible for maintaining homeostasis of the system and providing the capacity for repair and restoration after injury or stress. It is thought that some aberrant phenotypes within the hematopoietic system may be attributed to functional alterations of the stem cell compartment. Conversely, restoration of potential to dysfunctional HSCs may lead to long-term improvements of the hematopoietic system given HSCs’ unique self-renewal potential and ability to give rise to all cells within the hematopoietic system.

Here, we investigate the effects of supplementation of the NAD^+^ precursor NR on dysfunctional HSC compartments, from *Atm*^−/−^ and normal aged mice, to examine possible improvement of HSC potential. Our results showed significant alterations in lineage commitment of HSCs after NR treatment, with enhanced lymphoid potential. This correlated with changes in inflammatory cytokines and transcriptional alterations of the HSCs. While transplantation of aged NR-treated HSCs reproduced the enhanced lymphoid output seen in the transcriptional profiles, the changes in potential are not sustained after NR withdrawal in the aged mice, and the rebound phenotype appear to exacerbate aging phenotypes. Our results highlight the importance of the duration of NR exposure and the timing of initial exposure to derive robust, balanced lineage outputs from HSCs.

## Results

### NR supplementation increases lymphoid-progenitor cell frequencies in young *Atm*^−/−^ bone marrow

**Fig. 1:**
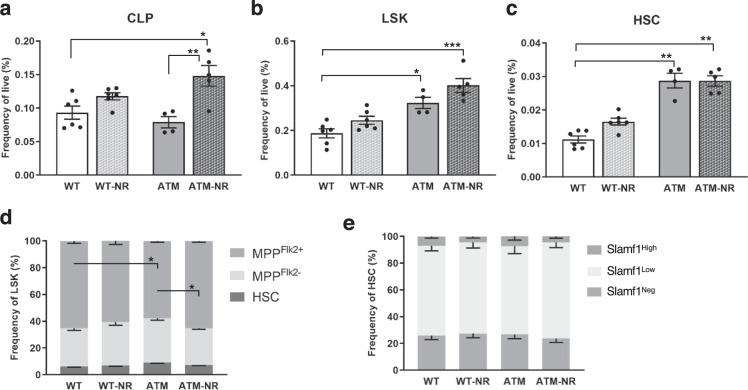
NR treatment increases lymphoid-progenitor cell frequencies in *Atm*^−/−^ bone marrow. **a**–**c** Frequency analysis of early progenitor compartments in NR-treated (NR) or untreated bone marrow from *Atm*^+/+^ (WT) or *Atm*^−/−^ (ATM) mice (2–3 months). CLP common lymphoid progenitors (Lin^−^IL-7Rα^+^Flk2^+^), LSK lineage^−^Sca1^+^c-Kit^+^, HSC Lin^−^c-Kit^+^Sca1^−^CD34^−^Flk2^−^. **d** Composition of the LSK compartment. **e** Frequencies of lineage-biased subsets of HSCs defined by Slamf1(CD150) expression. Data are represented as mean ± SEM. *n*: 4–6. Kruskal–Wallis test for **a**–**c**, two-way ANOVA for **d** and **e**. *q* value (Kruskal–Wallis test) or *p* value (two-way ANOVA): <0.05*, <0.01**, <0.001***.

Given the robust improvement in life and healthspan after NR treatment in models of A–T^1,11,15^, we first investigated the response of the hematopoietic system after supplementing *Atm*^−/−^ mice (ATM) and littermate *Atm*^*+*/+^ controls (WT) with a precursor of NAD^+^. Examination of hematopoietic progenitor cells in whole bone marrow (WBM) showed that NR-treated *Atm*^−/−^ mice had significantly increased common lymphoid-progenitor (CLP) frequency compared to the nontreated *Atm*^−/−^ mice (Fig. 1a and Supplementary Fig. 1). While NR treatment did not drive significant changes in the frequencies of the LSK (Lin^−^Sca1^+^cKit^+^) and HSC compartments of *Atm*^−/−^ mice, we did see significant aging-like phenotypes (increases) in these compartments in the *Atm*^−/−^ mice compared to WT (Fig. 1b, c). Consistent with the increased frequency of CLP in NR-treated *Atm*^−/−^ mice, MPP^Flk2+^ (including lymphoid-primed multipotent progenitors (LMPPs)^16^ and similar to MPP4^17,18^) frequency was also significantly increased compared to untreated *Atm*^−/−^ mice (Fig. 1d).

Changes of the lymphoid lineage progenitors in NR-treated *Atm*^−/−^ mice could be driven by differentiation choices of the earliest progenitor cells or through improved systemic environments supporting lymphoid-primed cell types. To determine if there were changes in the frequency of lineage-biased HSCs, we used CD150/Slamf1 to subfractionate the LSKCD34^−^Flk2^−^ cells^19^. We did not observe changes in the composition of the HSC compartment, with similar frequencies of balanced (Slamf1^Low^) and myeloid-biased (Slamf1^High^) HSCs both before and after NR treatment (Fig. 1e). However, the myeloid-biased HSCs contribute only modestly to the young WT and *Atm*^−/−^ HSC compartments, while the majority of HSCs already can robustly contribute to lymphoid output and NR effects may not be readily reflected in changes of these HSC compartments.

### NR supplementation improves HSC lymphoid potential in aged mice

**Fig. 2:**
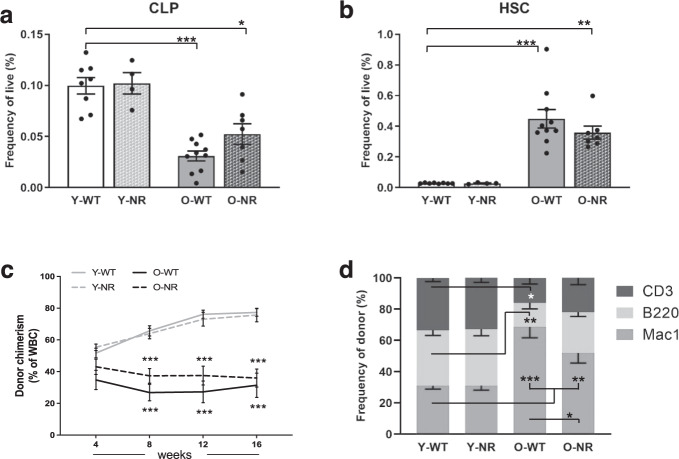
NR treatment improves the lymphoid potential of HSCs from aged mice. **a**, **b** Frequency analysis of early progenitor compartments in NR-treated (NR) or untreated bone marrow from young (Y-WT, 3–4 months) or aged (O-WT, 24–29 months) mice (*n* = 4–10). **c** Peripheral blood chimerism generated from 200 purified HSCs against 2 × 10^5^ competitor cells every 4 weeks for 4 months (*n* = 10). **d** Lineage composition of donor derived peripheral blood 4 months post transplant (*n* = 10). Data are represented as mean ± SEM. Kruskal–Wallis test for **a** and **b**, two-way ANOVA for **c** and **d**: <0.05*, <0.01**, <0.001***.

Given that *Atm*^−/−^ mice display many physiologic aging phenotypes and supplementing the *Atm*^−/−^ mice with NR led to increased lymphoid-progenitor cells (Fig. 1), we wanted to examine if NAD^+^ supplementation could have similar positive effects on aged wild-type mice. Analysis of progenitor cells from marrow of young and old C57BL/6J mice showed the expected decrease in frequencies of lymphoid progenitors (CLPs) in the old untreated mice (O-WT) compared to young mice (Y-WT)^20^. After NR treatment (O-NR) the CLP compartment frequency increased but did not return to levels in young mice (Y-WT) (Fig. 2a). NR treatment of old mice did not significantly decrease compartments that expand with age, including LSK (Supplementary Fig. 2a, b), HSC (Fig. 2b), or Slamf1^High^ (Supplementary Fig. 2c) cells, and NR did not show any effect on the young bone marrow compartments examined (Fig. 2 and Supplementary Fig. 2).

Though there were modest changes to the frequencies of the stem cells, we sought to address if NR supplementation affects the functional potential of HSCs from different ages with competitive transplants. Flow cytometric analysis of peripheral blood 4 months post transplant showed the expected decrease in old HSC donor contribution but there were no significant differences in chimerism derived from old HSCs after NR treatment (Fig. 2c). However, analysis of the lineage contribution from donor HSCs demonstrated old NR-treated HSCs have a more balanced lineage output, with a significant reduction in the frequency of the myeloid cells compared to untreated old HSCs (Fig. 2d and Supplementary Fig. 3).

### NR treatment reduces inflammation in old C57BL/6 mice

**Fig. 3:**
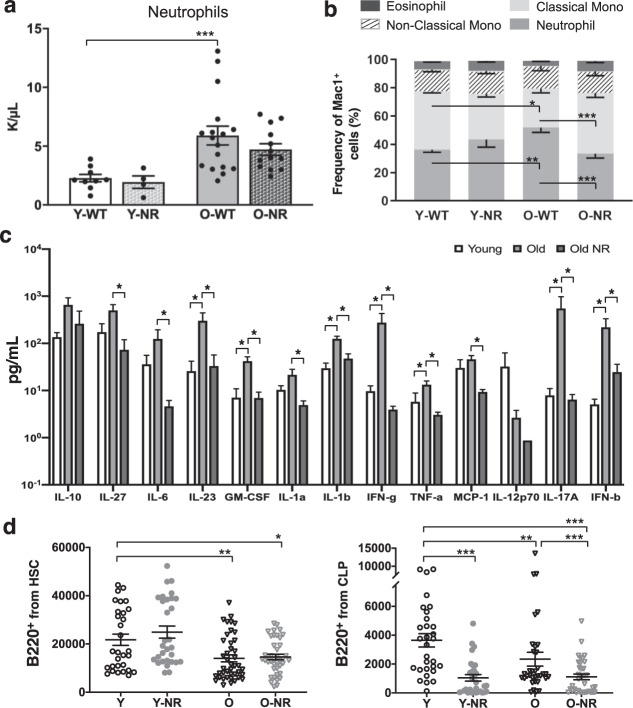
NR treatment reduces inflammation in old WT mice. **a** Complete blood cell count data from four experimental groups shows differences in neutrophils (*n* = 4–17). **b** Composition of Mac1^+^ cells from peripheral blood (*n* = 4–17). **c** Serum cytokine levels from young, old, and NR-treated old mice were evaluated with LEGENDplex^TM^ mouse inflammation panel (*n* = 6–7). **d** B220^+^ cells derived ex vivo from HSCs and CLPs. Data are represented as mean ± SEM. Kruskal–Wallis test for **a**, **c**, and **d**, two-way ANOVA for **b**. *q* value (Kruskal–Wallis test) or *p* value (two-way ANOVA): <0.05*, <0.01**, <0.001***.

To examine if the number of myeloid cells also decreases in the aged animals after NR supplementation, we examined complete blood cell counts in treated and untreated mice. The results showed the expected increase in neutrophils in the aged blood compared to young blood, but in aged, NR-treated mice there was a trend toward decreased neutrophil count (Fig. 3a). Examination of the distribution of myeloid lineage cells with flow cytometry showed a significant reduction in the frequency of neutrophils, yet an increase in inflammatory monocytes^21,22^ when comparing O-NR to O-WT mice (Fig. 3b).

Neutrophils and monocytes are implicated in the context of diverse inflammatory responses and also can be functionally involved though the intermediate of cytokine production^23,24^. Given the intimate association between inflammation, myeloid cells, and aging^23^, we wanted to examine if NR treatment in aged mice might affect inflammatory signals. Using a commercially available immunoassay of murine inflammatory cytokines, we show old mice have higher levels of inflammatory cytokines compared to young mice, with 7 of the 13 cytokines examined significantly elevated in circulation of aged mice. Notably, after NR treatment in old mice, there was a significant decrease in the levels of 11 of the 13 examined inflammatory cytokines compared to old untreated mice (Fig. 3c). These data suggest short-term NR treatment in inflamed environments, such as aged mice, leads to a significant reduction in inflammatory signals, corresponding to a decrease of granulocytes yet a significant increase in the inflammatory monocytes.

Previous studies have shown inflammatory cytokines such as IL-1β and TNFα can affect HSCs by promoting myeloid differentiation at the expense of lymphoid differentiation^25–28^, thus we wanted to examine if global decreases in inflammatory cytokines are necessary to drive alterations in lineage potential of the aged HSCs or if direct NR supplementation could drive lineage potential alterations seen when NR-treated HSCs were transplanted. To address this, we performed ex vivo culture assays with conditions promoting B-cell differentiation. Three independent experiments were performed with a minimum of 6 replicates per condition (average of 12 replicates). Analysis of total B220^+^ cells derived from young and aged HSCs showed reduced B220^+^ cell output from the aged HSCs compared to young HSCs (Fig. 3d); however, direct supplementation of NR in vitro did not contribute to significantly improved B-cell output from HSCs. Notably, in vitro supplementation of NR to CLPs had significantly negative effects on the B-cell output of both young and old cells (Fig. 3d).

### NR supplementation leads to transcriptional alterations in aged HSCs

**Fig. 4:**
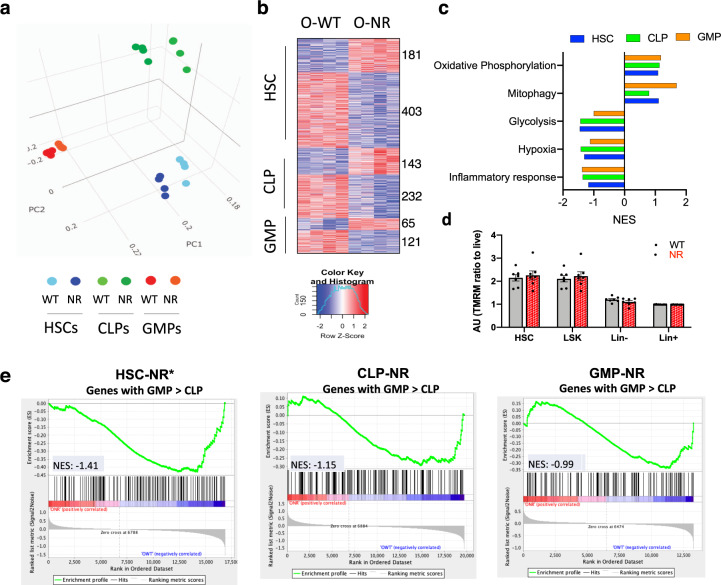
NR supplementation leads to transcriptional alteration in aged HSCs, CLPs, and GMPs. **a** PCA analysis of total RNA-seq transcripts from old WT and NR-treated HSCs (Blue), CLPs (Green), and GMPs (Red). **b** Heatmap analysis of differentially expressed genes in HSCs, CLPs, and GMPs (O-WT vs O-NR) determined using the following thresholds: expression levels CPM > 3, and *p* value < 0.01. **c** Normalized enrichment scores (NES) for gene sets comparing NR and untreated cells. **d** Quantification of ΔΨm in aged bone marrow populations after NR (with verapamil). **e** Gene set enrichment analysis (GSEA) for genes belonging GSE37301_CLP_VS_GMP_Down show decreased expression after NR supplementation in all HSC, CLP, and GMP populations.

Given the positive effects of improved lymphoid output appear to require additional endogenous signals and that different progenitor cells responded differently to NR ex vivo (Fig. 3d), we wanted to examine if there were cell-intrinsic alterations in the aged progenitors after NR treatment in vivo. Transcriptional changes were evaluated by generating total RNA sequencing (RNA-seq) datasets of HSC, CLPs, and granulocyte–monocyte progenitors (GMPs) isolated from old WT (O-WT) and NR-treated (O-NR) mice. Principle component analysis (PCA) showed that specific progenitors cells cluster together, supporting the reproducibility of the data (Fig. 4a). These data also showed each of the cell types had changes in their transcriptional landscape after NR treatment (Fig. 4a). The progenitor cells with the greatest differences in transcription were the HSCs (Fig. 4a, b). The majority of the significant transcriptional changes were decreases in expression in all progenitors: HSC-NR: 403, CLP-NR: 232, and GMP-NR: 121. Overall, the transcription of myeloid progenitors was least affected by the NR treatment (Fig. 4b and Supplementary Table 1). Gene set enrichment analysis (GSEA) revealed that the glycolysis pathway was repressed in all NR-treated progenitor cells (Fig. 4c), similar to human muscle cells after NR^29^. The upregulated oxidative phosphorylation and mitophagy pathways, together with downregulated hypoxia and inflammatory response pathways, indicate a reduced stress state^30^ (Fig. 4c), which is consistent with the observation that NR treatment reduces the level of inflammatory cytokines in aged mice (Fig. 3c). To address if these transcriptional changes correlated with differences in mitochondrial potential, we stained HSCs, LSKs, lineage negative (Lin^−^) and positive (Lin^+^) cells with TMRM, and the efflux pump inhibitor, verapamil. We saw HSCs had more robust mitochondrial potential than differentiated cells, but there were no significant alterations in potential after NR treatment in any population we assayed (Fig. 4d).

Looking for specific enrichment of pathways relevant to hematopoiesis, we found that after NR treatment the stem and progenitor cells decreased expression of genes robustly expressed in myeloid progenitor cells compared to lymphoid-progenitor cells (Fig. 4e and Supplementary Fig. 4, GSE37301_CLP_VS_GMP_Down). These data further support the intrinsic alterations of stem and progenitor cells contributing the shift from myeloid toward more lymphoid lineage after NR treatment.

### Alterations driven by NR supplementation are not sustained after NR withdrawal

**Fig. 5:**
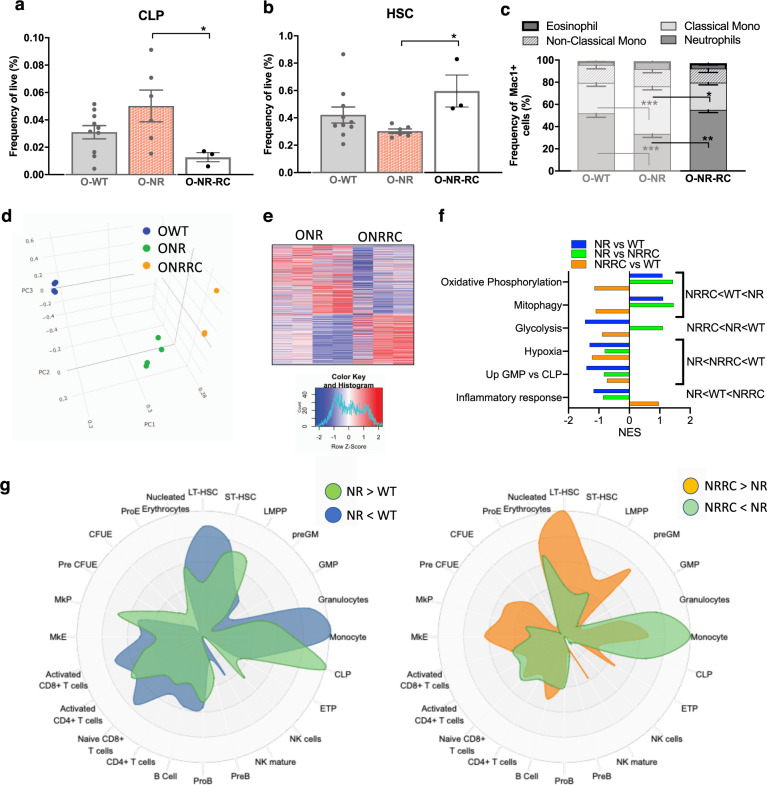
NR supplementation effects are not sustained upon NR removal. **a**, **b** Frequency analysis of early progenitor compartments in old mice after recovery (NR-RC: 2 months of supplement withdrawal) compared to O-WT and O-NR (*n* = 3–10). **c** Composition of Mac1^+^ cells from peripheral blood (*n* = 3–17). **d** PCA analysis of total RNA-seq transcripts from old WT (Blue) and old NR-treated HSCs (Green), and old NR-RC HSCs (Orange). **e** Heatmap analysis of differentially expressed genes in O-NR and after recovery (ONRRC) determined using the following thresholds: expression levels CPM > 3, and *p* value < 0.01. **f** Normalized enrichment scores (NES) for gene sets comparing old-WT, old-NR, and old-NRRC HSCs. **g** CellRadar plot indicating the lineage potential derived from transcriptional changes between old WT and old NR (left: enriched in NR, green/depleted in NR, blue) and between old NR and old-NRRC (right: enriched after NR removal, orange/depleted after NR removal, green). Data are represented as mean ± SEM. Kruskal–Wallis test for **a** and **b**, two-way ANOVA for **c**. *q* value (Kruskal–Wallis test) or *p* value (two-way ANOVA): <0.05*, <0.01**, <0.001***.

Given the improved lymphoid output in NR-treated aged HSCs, we also wanted to evaluate if these NR-driven phenotypes could be sustained after NAD^+^ supplement removal in an aged environment. We analyzed old mice treated with NR that received a 2-month washout period. NR supplement-directed changes in frequency seen in the primitive compartments (CLP and HSC) were significantly reversed after withdrawal of NR (O-NR-RC: Fig. 5a, b). The same rebounding effect was also seen in the myeloid cell compartment of the peripheral blood (Fig. 5c), in which the neutrophil compartment (reduced after NR treatment) becomes the predominant population after NR withdrawal. Given these striking reversals of progenitor cell and peripheral blood composition after supplement removal, we purified HSCs from the old NR-RC mice and performed RNA-seq analysis. PCA clustering among the three groups of isolated HSCs shows the O-WT separate furthest away from both the O-NR and O-NR-RC groups (Fig. 5d). Further, the O-NR-RC HSCs do not revert back toward the O-WT HSCs on two main component axes (PC1 or PC2), but instead move away from O-NR HSCs in a different trajectory. We found 107 significantly upregulated and 126 significantly downregulated transcripts between O-NR and O-NR-RC HSCs (Fig. 5e). Pathways previously implicated in NR benefits and examined in Fig. 4c were evaluated in HSCs after the removal of NR (Fig. 5f). We see in these pathways the NR-RC HSCs do not revert back toward WT expression. For both OxPhos and mitophagy, after NR, there is an enhancement of transcripts involved in those pathways. After removal of NR, there is decreased expression of these pathways, and reversion of expression is to an extent that is even lower that what was seen originally in old-WT HSCs (NRRC < WT < NR). Similarly, the increased expression of genes involved in the inflammatory pathway in O-NR-RC HSCs overshoot the expression levels of these genes in old WT HSCs (NR < WT < NRRC). NR-RC transcripts involved in hematopoietic specific comparisons (GMP vs CLP) as well as hypoxia were at intermediate levels, between the NR and WT conditions (NR < NRRC < WT). Finally, for genes associated with glycolysis, instead of reversion toward or beyond the expression levels in the WT HSCs, their expression in O-NRRC HSCs is even lower than in the NR-treated old HSCs (NR-RC < NR < WT). These data suggest that removal of the supplement does not lead to direct reversion back toward untreated states, but instead may have exaggerated negative effects on the hematopoietic system.

To examine hematopoietic signatures in the NR-treated HSC and after NR removal, we used CellRadar^31^ to compare genes up- and downregulated in the HSC transcriptomes against published lineage associated genes. After NR treatment, compared to WT, the NR HSCs are enriched for profiles associated with lymphoid-progenitor cells including CLPs and LMPPs, while depleted for profiles associated with HSCs or myeloid cells (Fig. 5g). These expression profiles mirror the lineage output of the NR-treated mice as well as the change in lineage potential during HSC transplant paradigms (Fig. 2). After the washout period, the NRRC HSCs have a more HSC-centric profile and elevated expression of genes associated with myeloid and erythroid progenitor populations (Fig. 5g). The NRRC HSCs also show repressed monocyte signatures (Fig. 5g), which corresponds with cell composition changes seen in the hematopoietic system after withdrawal of the supplement (Fig. 5a–c). Together, these data suggest that cell-intrinsic alterations contributing to improved lymphoid potential in the aged environment are not sustained after NR withdrawal, and that withdrawal in an aged environment may lead to more severe myeloid skewing compared to normal aging.

## Discussion

The improved lymphoid lineage potential in mice with compromised hematopoietic systems after NR treatment suggests that elevated NAD^+^ levels could provide a more favorable systemic environment for these cells and/or promote differentiation toward lymphoid cell types. In both the *Atm*^−/−^ and aged environments, there is an associated loss of NAD^+^ levels compared to young, healthy mice^32,33^, and global administration of the NR likely has a direct effect not only on stem cells, but on all hematopoietic cells. In anecdotal data, NAD^+^ levels were elevated in bulk bone marrow cells after oral administration at concentrations used in our experiments. Of note, many hematopoietic cells express the NAD^+^ glycohydrolases CD38, CD157 (BST-1), and RT-6 (with robust expression on many lymphoid cell types)^34–36^ and elevating circulating levels of NAD^+^ may improve substrate availability to those cells. However, improved environmental stimuli cannot be the only contributing factor driving the altered lineage potential seen in the impaired systems after NAD^+^ supplementation, as transplants of purified HSCs from the NR-treated aged mice show long-term phenotypes of improved lymphoid output (Fig. 2d).

While investigating the environmental contribution to the altered lineage potential, we detected a general decline in inflammatory cytokines in the circulation of NR-treated old mice (Fig. 3c). Of particular interest, regarding the function of HSCs, is the reduction of the IL-1 cytokine level, which has been shown to play a critical role in driving HSC myeloid differentiation^28^. This decline in myeloid differentiation potential may be contributing to the decreased numbers of neutrophils (Fig. 3a, b). As neutrophils are mediators of inflammation, responding to signals and producing cytokines and other inflammatory factors regulating inflammation^37,38^, reduction of these cells would dampen the feedback loop of increased inflammatory cytokines and increased myeloid cell production. However, this decrease in neutrophils is also associated with an increase in frequency of inflammatory monocytes, but CBC data show the numbers of monocytes remains lower in NR-treated mice (data not shown). This global decrease in myeloid differentiation could then allow for increased frequencies of lymphoid progenitors and decreased overall inflammation, mitigating two major negative features in aged hematopoiesis. We also noted that the decline in inflammatory factors we see after short-term NR supplementation in both the aged (Fig. 3c) and *Atm*^−/−^ mice^39^ overlapped with those reported (IL-6 and TNF-α) in a study of oral NR supplementation in humans^29^.

We were unable to reproduce the improved lymphoid potential of either HSCs or CLPs with direct exposure to NR in ex vivo culture conditions (Fig. 3d) though the improved lymphoid potential of old HSCs was maintained in transplanted young, irradiated recipients (Fig. 2d). This is possibly due to suboptimal differentiation conditions ex vivo or the inability to reproduce the systemic changes of NR supplementation that may contribute to the altered potential of the primitive cells. To characterize the lasting effects of NR exposure in the aged mouse, we provided a 2-month washout. We saw that the improved lymphoid output seen after NR was not sustained in the aged environment after supplement removal. Together, this suggests that the transcriptional changes of the progenitor cells are augmented by environmental stimuli—associated either with a young-recipient environment or NAD^+^ supplemented aged environment—and the combination is required to sustain the improved lymphoid lineage output.

Using transcriptome analysis to address changes in progenitor cells that could contribute to functional alterations in the treated HSCs, we did not see striking differences in genes implicated in other studies of NAD^+^ repletion, such as the sirtuin genes, *Parp1*, or even *Cd38* when comparing NR-treated old HSCs to untreated old HSCs (data not shown). We also did not see significant changes in overall mitochondrial DNA content after NR treatment (data not shown) or in mitochondrial membrane potential associated with the improved functional potential of HSCs after NR (Fig. 4d). One potential caveat is that we did not directly measure HSCs’ mitochondrial function, and we did see a minor increase in the expression of *PGC-1α*, which encodes the protein PGC-1α, a master regulator of mitochondrial biogenesis, in O-NR HSCs compared to O-WT. This expression change, however, did not reach our minimal significance threshold of FDR < 0.05. Since NAD^+^ coordinates both mitochondrial biogenesis (e.g., via PGC-1α) and the elimination of damaged mitochondria via mitophagy, it may lead paradoxical reports on either increased or reduced mitochondrial content by NAD^+^ in different conditions^2,4,40^.

Though we see improvement after NR supplementation in the deficient (*Atm*^−/−^ or old) HSCs, we did not see the recently reported improved HSC potential after 1 week of NR treatment in young mice^41^. These differences could be due, in part, to the duration of treatment, the vehicle for administration of NR, gender differences (male vs female), and potentially background strain differences between the groups. Thus, it will be important to further examine some of these key differences, with particular focus on duration of NR treatment and effects on robust versus compromised hematopoietic environments.

While more than seven phase I clinical trials have shown NR supplementation drives elevated NAD^+^ levels in the blood and is well tolerated in middle and old aged individuals^4,42^, potential translation of benefits from NAD^+^ supplementation in human hematopoiesis remain unresolved. Our data suggest that NR administration in compromised hematopoietic systems (NAD^+^ depleted system) drives cell-intrinsic changes of the HSC compartment together with an overall reduction in circulating inflammatory cytokines. However, once the aged system has been exposed to NR, the effects of removing the supplementation may have undesirable consequences. Thus, once a deficient system is exposed to NAD^+^ supplementation, to maintain the benefits we have demonstrated, the regimen may need to be sustained long-term.

## Methods

### Mice

The ATM heterozygous strain (B6;129S4-Atmtm1bal/J) was purchased from The Jackson Laboratory and bred at the NIA facility. After weaning, *Atm*^−/−^ mice and *Atm*^+/+^ littermates were given NR (12 mM) from ChromaDex in drinking water. Mice used in the aging studies were C57BL/6 males: young (3–4 months) and old (24–29 months). NR was supplemented for 4–6 weeks^11^. In condition of NR supplementation, water bottles were changed twice a week. All animal experiments were performed under protocols approved by NIA Institutional Animal Care and Use Committees. Transplant recipient and competitor cells were young female B6.SJL-Ptprca Pepcb/BoyJ (CD45.1) from The Jackson Laboratory.

### Purification of cells

For HSC sorting, bone marrow was first selected as c-Kit positive (PE-cKit antibody and EasySep™ PE Positive Selection Kit II). Enriched cells were stained with antibodies against lineage (Ter119 (BioLegend, 116232, 1/200), B220 (BioLegend, 103227, 1/200), Mac1 (BioLegend, 101224, 1/200), CD3 (BioLegend, 100214, 1/200), Gr1 (BioLegend, 108434, 1/200), IL-7Rα (BioLegend, 135024, 1/200)), cKit (BioLegend, 105808, 1/200), Sca1 (BioLegend, 108126, 1/200), CD34 (eBioscience, 11-0341-85, 1/50), Flk2 (BioLegend, 135310, 1/50), CD150 (BioLegend, 115914, 1/200), and PI and sorted as PI-Lin-cKit+Sca1+CD34-Flk2-CD150+.

### Transplantation experiments

A total of 200 HSCs pooled from donor mice were transplanted into lethally irradiated (9.56 Gy) recipients against 2 × 10^5^ competitor (CD45.1) WBM cells. Peripheral blood analysis was performed at 4-week intervals post transplantation.

### Peripheral blood analysis

CBC data collected using a Hemavet 950FS and frequencies of populations were generated using flow cytometry after ACK treatment and staining with for Ter119 (BioLegend, 116228, 1/100), B220 (BioLegend, 103224, 1/100), Mac1 (BioLegend, 101216, 1/100), CD3 (BioLegend, 100244, 1/100), and Gr1 (BioLegend, 108406, 1/100) and PI (Supplementary Table 2). CD45.1 (BioLegend, 110714, 1/100) and CD45.2 (BioLegend, 109820, 1/100) antibodies were included in transplant blood analysis.

### WBM analysis

Bone marrow cells were ACK treated and then stained with antibodies Ter119 (BioLegend, 116204, 1/100), B220 (BioLegend, 103204, 1/100), Mac1 (BioLegend, 101204, 1/100), CD3 (BioLegend, 100244, 1/100), Gr1 (BioLegend, 108404, 1/100) (Lin), cKit, Sca1, CD34, Flk2, CD150, IL-7Rα FcγRα (eBioscience, 45-0161-82, 1/100), and PI (Supplementary Table 2). Biotinylated antibodies were stained using Streptavidin-Pacific Orange (Thermo Fisher Scientific) before staining PI. Posttransplant marrow was stained with antibodies Ter119, B220, Mac1, CD3, Gr1, IL-7Rα (Lin), cKit, Sca1, CD34, Flk2, CD150, FcγRα, CD45.1 (BioLegend, 110728, 1/100), CD45.2, and PI.

### Serum cytokine assays

Blood was collected by cardiac puncture at the time of sacrifice. After 15 min room temperature incubation, samples were centrifuged at 500 × *g* for 15 min at 4 °C. The sera were collected and stored at −25 °C. Serum cytokines were assayed using LEGENDplex^TM^ (BioLegend) according to the manufacturer’s instructions. For data acquisition and analysis, a CytoFLEX (Beckman Coulter) and LEGENDplex v.8 data analysis software were used, respectively.

### Ex vivo culture analysis

A total of 100 HSCs were cultured in 0.2 mL of αMEM supplemented with 10 ng/mL of Flt3L, 5 ng/mL of IL-3, 20 ng/mL of IL-7, 50 ng/mL of SCF, 100 mM of β-MEM, 20% of FBS, and NR (0.0 or 2.5 mM) in 96-well flat bottom plates precoated with OP9 cells (ATCC) at 4 × 10^4^ cells/well. A total of 100 CLPs were cultured in 0.2 mL of ExVivo15 with 100 ng/mL of Flt3L, 20 ng/mL of IL-7 and SCF, 50 mM of β-MEM, 1% of BSA, and NR (0.0 or 2.5 mM) in 96-well round bottom plates. After 48 h with NR, the media were replaced with fresh media excluding NR. The media were replaced every 3–4 days. After 14 days (HSC) or 10 days (CLP) of culture, cells were analyzed by flow cytometry for the differentiation to B cells (B220^+^). Culture plates were maintained at 5% CO_2_ and O_2_ at 37° C. Recombinant mouse cytokines were purchased from Peprotech.

### Mitochondrial membrane potential assay

Sorted cells were incubated with 2 nM tetramethylrhodamine, methyl ester, perchlorate (TMRM) in StemSPAN SFEM supplemented with 50 ng/mL SCF and TPO, and 50 µM verapamil for 60 min at 37° C followed by a wash. PI with 2 nM TMRM was used for dead cell exclusion and data were collected on a FACSAria Fusion. Flow data analysis was performed using FlowJo.

### RNA-seq and analysis

RNA was purified with TRIzol™ Reagent and Direct-zol RNA Microprep. RNA-seq libraries were constructed using SMARTer Stranded Total RNA-Seq Kit v2 - Pico Input Mammalian according to the manufacturer’s protocol. Sequencing was done on Illumina HiSeq 2500 instrument using 2 × 105 bp reads.

A total of 2.2 billion sequencing reads were used, with an average of 62 million reads per sample. For analysis of transcriptome datasets, we built an index sequence for STAR using the Gencode M22 reference feature including protein-coding and noncoding genes. Prior to sequence alignment, we applied trim galore (version 0.4.3) with cutadapt package (version 1.12)^43^ to remove any unnecessary genomic fragments (e.g., adapter dimers) and low-quality nucleotide sequences from the raw reads. We mapped adapter trimmed sequencing reads to the mouse reference genome (mm10) using STAR aligner^44^ and calculated the raw count using featureCounts package (gene level)^45^. Differentially expressed transcripts (DETs) lists were generated with edgeR using cutoff: fold change > 1.5, CPM > 3, FDR < 0.05, or *p* value < 0.01^46^. GSEA was performed for pathway analysis^47^. Visualization of the DET profiles in HSCs was performed using software CellRadar^31^.

### Statistical analysis

Prism software (GraphPad) was used for statistical analyses. Statistical analysis used in this study was Kruskal–Wallis test or two-way ANOVA. Data are presented as mean ± S.E.M. and *q* value (Kruskal–Wallis test) or *p* value (two-way ANOVA) < 0.05 were the minimal threshold for statistical significance. The statistical parameters and number of samples can be found in the figure legends.

### Reporting summary

Further information on research design is available in the Nature Research Reporting Summary linked to this article.

## Supplementary information

Supplementary Information

Reporting Summary

## Data Availability

RNA-seq data are available on GEO with accession number GSE147662.

## Code availability

No new code was generated or used in this manuscript and RNA-seq analysis code is publicly available at https://github.com/Zhang-lab/TaRGET-II-RNAseq-pipeline.
