# Supplementary material for: NAD+ augmentation with nicotinamide riboside improves lymphoid potential of Atm−/− and old mice HSCs
Source: NPJ Aging Mech Dis. 2021 Sep 21;7:25. doi: 10.1038/s41514-021-00078-3 (PMC8455618; doi:10.1038/s41514-021-00078-3)
Supplement: Supplementary file 1 — Supplementary Information [file 41514_2021_78_MOESM1_ESM.pdf]

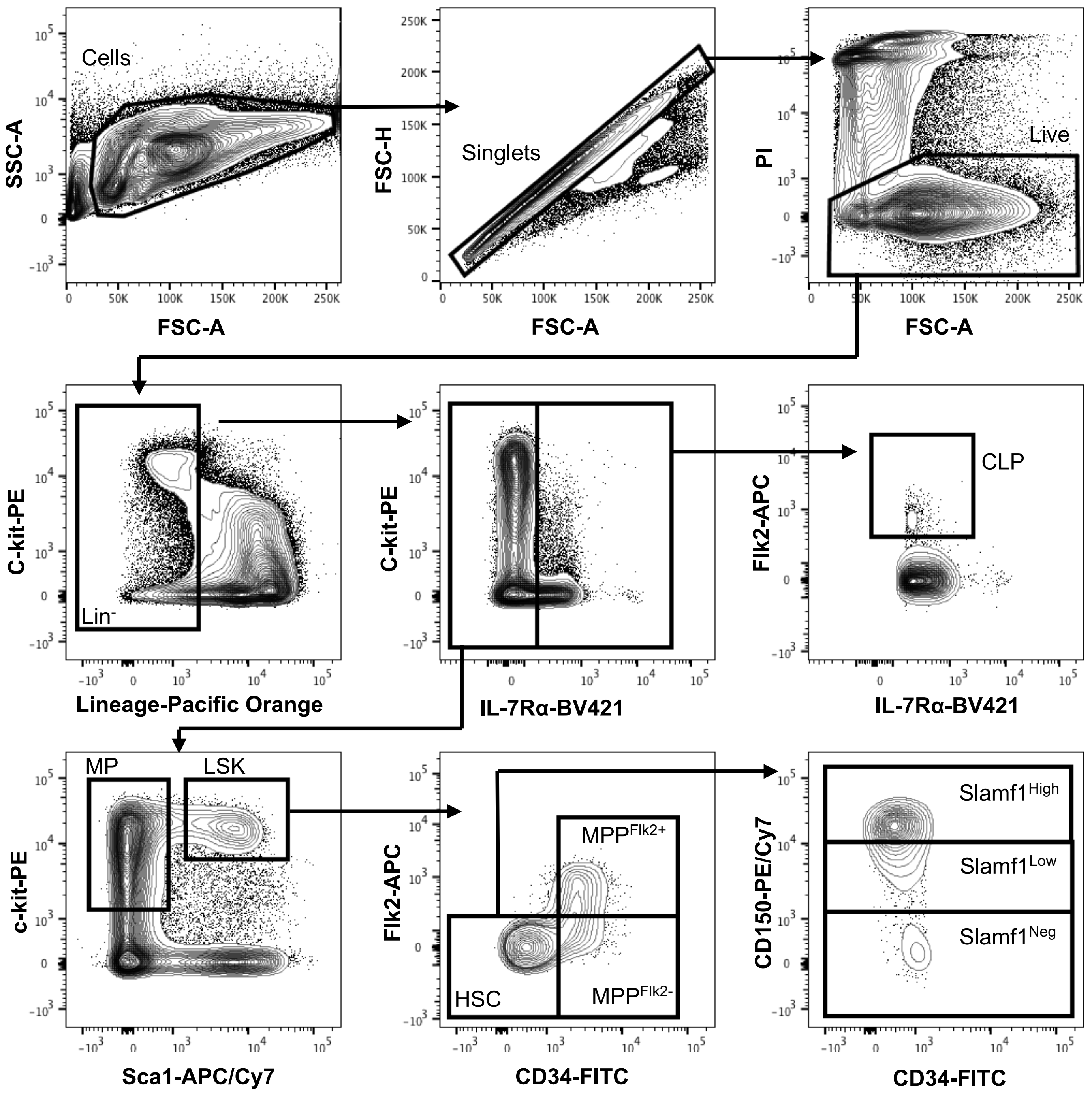

Supplementary Fig. 1. Gating strategy of whole bone marrow analysis. Representative gating strategy of populations gated in whole bone marrow analysis associated with Figures 1,2, and 5. **CLP**: Figure 1a, 2a, and 5a. **LSK**: Figure 1b, Supp Figure 2a **HSC:** Figure 1c,d, 2b, and 5b, and Supp Figure 2b **MPPFlk2^{-} and MPPFlk2^{+}** : Figure 1d and Supp Figure 2b, **Slamf1^{High}, Slamf1^{Low}, Slamf1^{Neg}**: Figure 1e and Supp Figure 2c.

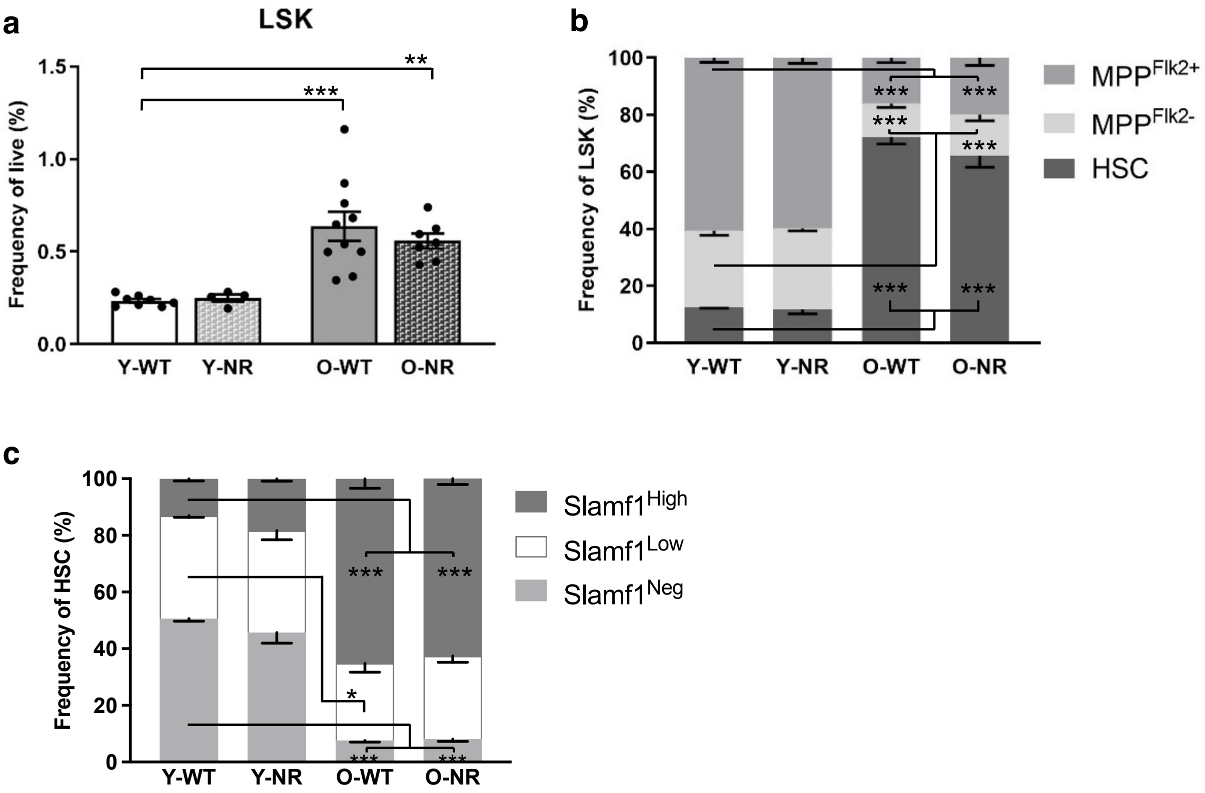

Supplementary Fig. 2. WBM analysis of young and old mice treated with NR. (a) Frequency analysis of LSK in NR-treated (NR) or untreated bone marrow from young (Y-WT, 3-4 months) or aged (O-WT, 24-29 months) mice (n = 4-10). (b) Composition of the LSK compartment (n = 4-10). (c) Frequencies of lineage-biased subsets of HSCs defined by Slamf1(CD150) expression (n = 4-10). Data are represented as mean ± SEM. Kruskal-Wallis test for Fig. a, two-way ANOVA for Fig. b-c. q-value (Kruskal-Wallis test) or p-value (two-way ANOVA): < 0.05 *, < 0.01 **, < 0.001 ***

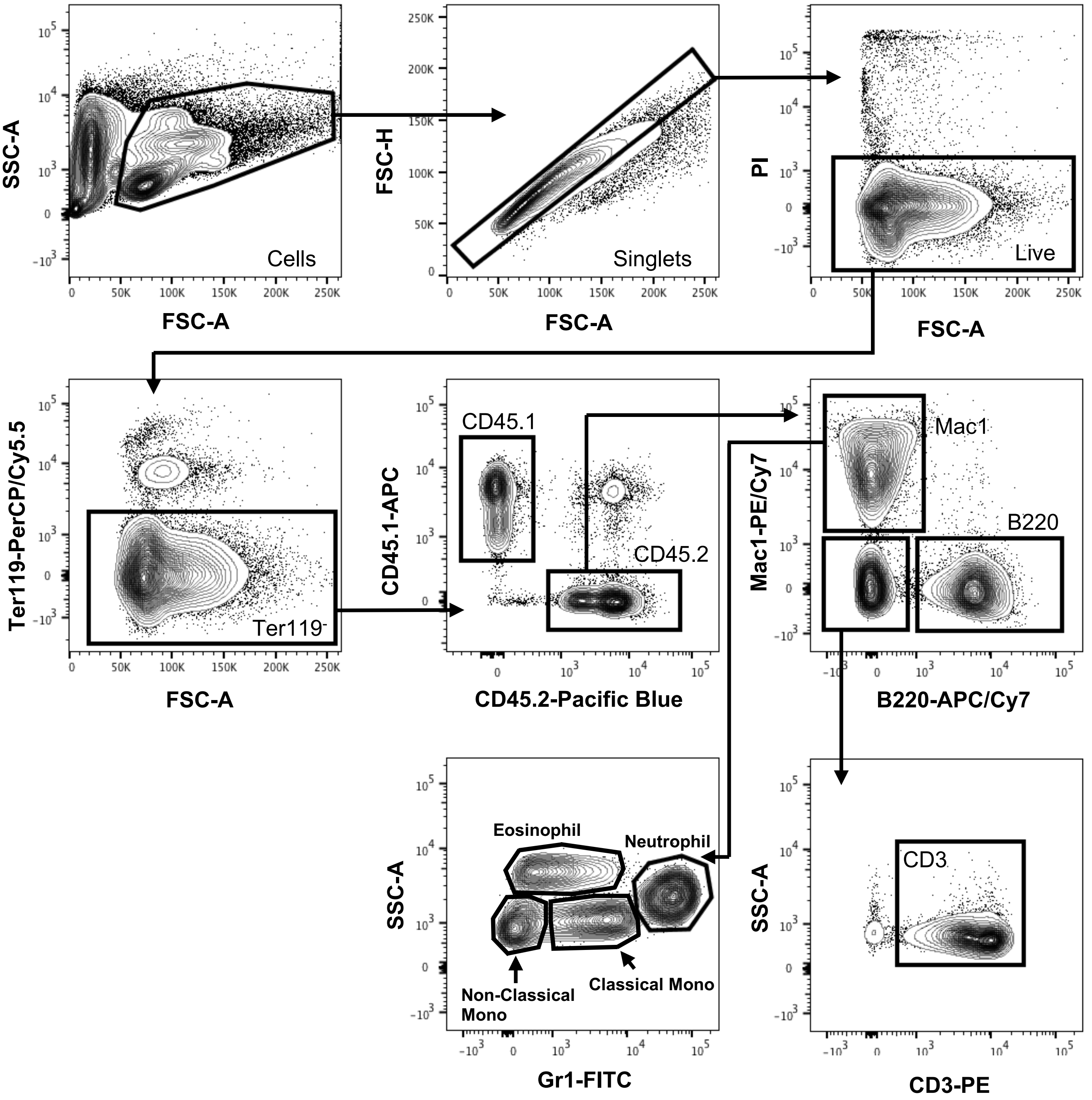

Supplementary Fig. 3. Gating strategy of peripheral blood analysis. Representative gating strategy of peripheral blood analysis in Figures 2,3, and 5. **CD45.2:** Donor chimerism Figure 2C, **CD3, B220, Mac1:** Figure 2d, **Eosinophil, Neutrophil, Classical Mono, Non-Classical Mono**: Figure 3b and 5c

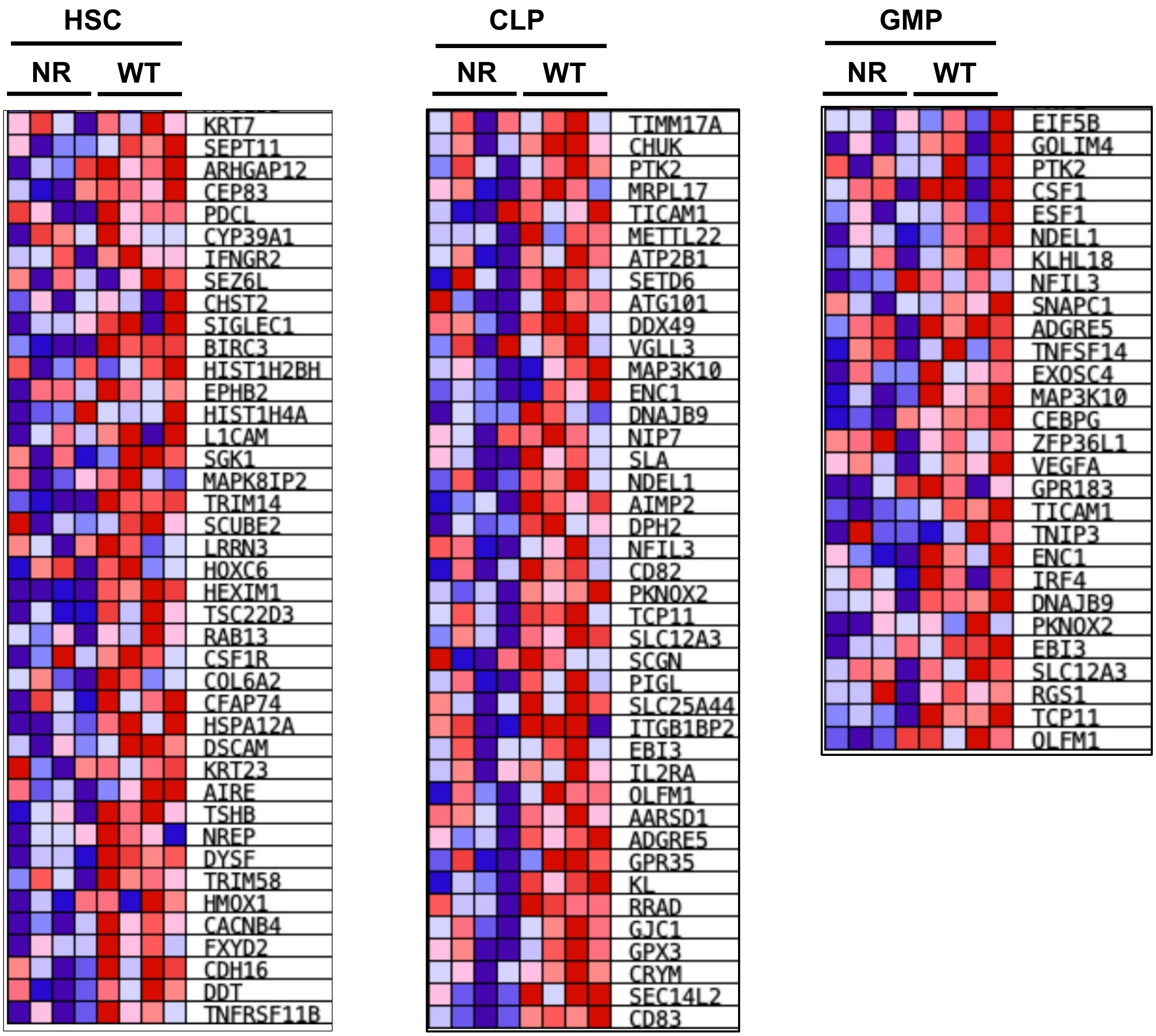

Supplementary Fig. 4. Heatmap of major downregulated genes enriched in Figure 4e. Expression of genes with differential expression driving belonging GSE37301_CLP_VS_GMP_Down in all HSC, CLP, and GMP populations.

Supplementary Table 1: DEGs of HSC, CLP, and GMP cells after NR treatment

Gene Name	log2FC(pos up in NR, neg down in NR)	P-value	Cell type
Car1	2.307532493	0.00504435	HSC
Gm38220	0.972794541	4.13E-06	HSC
4930548J01Rik	0.969472065	0.000355202	HSC
P2ry10	0.944270482	0.000127591	HSC
Art2b	0.875725931	0.000399242	HSC
Sema4c	0.872605622	6.38E-06	HSC
Gm18341	0.868509487	0.000382122	HSC
Gm26637	0.839110353	0.002582883	HSC
Gm15879	0.8320869	0.001289659	HSC
Zfp990	0.820321805	0.000258207	HSC
Gm3912	0.813761619	0.004463712	HSC
Dhrs3	0.806308536	2.02E-05	HSC
Arsg	0.79750366	0.000311932	HSC
Lrrc74b	0.788247323	0.001190071	HSC
Gm28856	0.78453949	0.000898816	HSC
Gm26982	0.769241881	0.002026593	HSC
Hrasls5	0.75864051	0.004586533	HSC
Pcdhb18	0.752837352	0.001263824	HSC
Gm15978	0.744310986	0.001707277	HSC
Mmp21	0.743066462	0.006499983	HSC
Inpp5j	0.741928948	0.009059199	HSC
Cartpt	0.741248537	0.001872857	HSC
Slc1a4	0.735362147	0.000109614	HSC
9330162012Rik	0.735260335	0.004073445	HSC
Mettl7b	0.733420548	0.001982193	HSC
Gm30698	0.72221051	0.001313411	HSC
Car9	0.721731017	0.000562223	HSC
Gm17809	0.719423804	0.002223929	HSC
Cyp3a63-ps	0.719009081	0.003315963	HSC
Ass1	0.717118653	0.000240408	HSC
Gm18913	0.708552697	0.004425759	HSC
Gpa33	0.706246968	0.007899153	HSC
Foxn1	0.70452302	0.007823876	HSC
Meig1	0.692722994	0.00925135	HSC
2610318N02Rik	0.692319526	0.000100995	HSC
Nox4	0.685711044	0.001646652	HSC
Gdf3	0.678840465	0.007815949	HSC
C430019N01Rik	0.677677148	0.00562641	HSC
Trabd2b	0.670740145	0.001836036	HSC
Epdr1	0.663424792	7.89E-05	HSC

4933427D06Rik	0.661144665	0.002814724	HSC
Dpf1	0.654272091	0.006336166	HSC
Gm37469	0.646106505	0.008906567	HSC
Gm11423	0.643310021	0.008568287	HSC
Tmem117	0.642393514	0.004193632	HSC
Cirbp	0.642059161	1.88E-05	HSC
9130230N09Rik	0.633185752	0.004694521	HSC
Gm28512	0.628223647	0.005396099	HSC
Gm33045	0.626549524	0.007801165	HSC
Aph1c	0.62544098	0.000655457	HSC
BC021767	0.625154613	0.006623705	HSC
Nupr1l	0.622860149	0.001818978	HSC
Gm44210	0.619882007	0.000683401	HSC
H3f3aos	0.617633885	0.003273886	HSC
Gm15318	0.6155043	0.005134637	HSC
Fam189a2	0.615239658	0.002912669	HSC
Wee2	0.613366164	0.007929184	HSC
4930509G22Rik	0.605854636	0.00462623	HSC
Trib2	0.599387621	0.003622397	HSC
Tmem86a	0.596724003	0.003108359	HSC
6030445D17Rik	0.595269496	0.004452295	HSC
Fam221a	0.595060619	0.002007222	HSC
Ovgp1	0.592747973	0.00815043	HSC
Mettl1	0.591128672	0.000772013	HSC
Tmem218	0.589495821	0.000475107	HSC
Cdkn2c	0.581935516	0.007888787	HSC
Mocos	0.5717957	0.000426675	HSC
C230035I16Rik	0.571236526	0.007988641	HSC
Gm38376	0.570737526	0.008243918	HSC
Gm32051	0.565858675	0.007420382	HSC
Ubox5	0.563538178	0.001145438	HSC
Gm36738	0.562343215	0.005328338	HSC
Gm16124	0.561120831	0.00691028	HSC
Hyal2	0.55550034	0.002451165	HSC
Gm26559	0.554781153	0.007212964	HSC
Gm37420	0.551136801	0.00573317	HSC
Slc7a5	0.546607459	0.000115155	HSC
B130034C11Rik	0.545683386	0.006027563	HSC
Zbtb25	0.540073629	0.00104831	HSC
Arrdc3	0.531796065	0.00599056	HSC
Sdr42e1	0.528368242	0.004556597	HSC
Gm17586	0.52809222	0.003544731	HSC
Gm44168	0.509712389	0.001870351	HSC

Gm13375	0.504263775	0.002843003	HSC
Thbd	0.496779007	0.004689077	HSC
Pccb	0.494507139	4.61E-05	HSC
AU041133	0.492116203	0.007700582	HSC
Gm37558	0.491844192	0.002072287	HSC
2610301B20Rik	0.490916293	0.009259564	HSC
Gm44037	0.487396772	0.006758629	HSC
Acadsb	0.484196105	0.002219846	HSC
Fbxo16	0.480566103	0.00993977	HSC
Pcgf1	0.480209599	0.003153902	HSC
5730409E04Rik	0.479198749	0.001525103	HSC
Mn1	0.478286067	0.006544754	HSC
Rsad2	0.477583704	0.000355259	HSC
Gm18194	0.476146765	0.002729966	HSC
Blm	0.464227526	0.00011444	HSC
C030034I22Rik	0.45998939	0.009407991	HSC
Cyp20a1	0.459903223	0.001295881	HSC
Otud1	0.455257526	0.005059253	HSC
Rhbdd1	0.451918675	0.000394601	HSC
Bcap29	0.451003776	0.000661267	HSC
Lhpp	0.440167738	0.005476262	HSC
Lnpk	0.4335389	0.00177182	HSC
Tm2d3	0.430453904	0.007243827	HSC
2810403D21Rik	0.428830512	0.006854139	HSC
Bend3	0.425470471	0.000256281	HSC
Ino80c	0.422889436	0.005175512	HSC
Rabl3	0.420331608	0.00414163	HSC
Cnot9	0.419934686	0.005914538	HSC
Paqr7	0.418720888	0.001733185	HSC
Proser1	0.417065913	0.001660899	HSC
Mri1	0.415371018	0.002196437	HSC
Arl2bp	0.413746423	6.80E-05	HSC
Sox12	0.412242322	0.006037908	HSC
Gm12216	0.411583531	0.007179092	HSC
Mtx3	0.411488415	0.000951972	HSC
Zfp639	0.407481756	0.005693644	HSC
Zfp955b	0.406886307	0.002674915	HSC
Gm44731	0.406699416	0.00529123	HSC
Slc24a5	0.406631527	0.001936876	HSC
Med16	0.40569943	0.00095005	HSC
9930014A18Rik	0.40515338	0.001852091	HSC
Slc16a1	0.397012518	0.00525605	HSC
Slc43a2	0.392888348	0.007476361	HSC

Apobec1	0.392144765	0.007067298	HSC
Hexb	0.388848607	0.005374456	HSC
Spred2	0.387696036	0.000225396	HSC
Casc4	0.381254261	0.004354381	HSC
Rnf170	0.38097802	0.008072702	HSC
Galk1	0.376789453	0.007939202	HSC
Wdr73	0.372384175	0.003128683	HSC
C1ra	0.372110274	0.002893311	HSC
Rapgef3	0.367157846	0.001594791	HSC
Oxsm	0.361692745	0.009428545	HSC
Bckdhb	0.361379364	0.009897	HSC
Tmx2	0.359481153	0.005856529	HSC
Hnrnpd	0.358174108	0.000629094	HSC
Pdk1	0.357283702	0.000443984	HSC
Gstcd	0.356241211	0.006872067	HSC
Inafm1	0.355292884	0.00635344	HSC
Osgin1	0.353468178	0.007337971	HSC
Lgals3bp	0.351623867	0.000481091	HSC
Pcdh7	0.34956902	0.006290368	HSC
Rsad1	0.347454587	0.006662615	HSC
Hprt	0.338092983	0.006090339	HSC
H2-DMb1	0.337773101	0.00690018	HSC
Dctd	0.334772733	0.009020697	HSC
Mansc1	0.33297822	0.003199876	HSC
Zfp52	0.332560108	0.002250833	HSC
mt-Nd1	0.330437136	0.007876394	HSC
Spire1	0.329604173	0.004258387	HSC
Sfxn1	0.329025415	0.000309896	HSC
Fus	0.327220815	0.001158323	HSC
Slc19a1	0.324863311	0.008253574	HSC
Tspan14	0.320747058	0.002305516	HSC
Atic	0.319065573	0.009575534	HSC
Unk	0.31714803	0.005703496	HSC
Cysltr2	0.316190432	0.004958037	HSC
Dalrd3	0.315184026	0.001653694	HSC
Zdhhc8	0.313974431	0.009895215	HSC
Orc5	0.313203709	0.008565092	HSC
Fxyd5	0.311873786	0.004018943	HSC
5730455P16Rik	0.310379651	0.005879568	HSC
Vamp1	0.309931989	0.002894461	HSC
Nsun2	0.308137355	0.000179154	HSC
Zfp971	0.307934103	0.006994452	HSC
H2-DMa	0.306903308	0.006590118	HSC

Hnrnpdl	0.304908382	0.003903681	HSC
2410002F23Rik	0.297475841	0.007792684	HSC
Arhgap35	0.292477488	0.003979143	HSC
Sipa1	0.286927921	0.002000802	HSC
Tm7sf3	0.283065615	0.009188754	HSC
Tspan4	0.28106297	0.009924385	HSC
Tmem250-ps	0.280901851	0.006583265	HSC
Cd24a	0.273574581	0.000455881	HSC
Ankrd28	0.271920311	0.006901179	HSC
Carm1	0.271786757	0.009854809	HSC
Git1	0.270805754	0.004661132	HSC
Mapk1ip1l	0.266115715	0.006408773	HSC
Trim25	-0.270057447	0.003202771	HSC
Ipo13	-0.273357346	0.00941334	HSC
Cep350	-0.275732364	0.00691907	HSC
Atp6v1d	-0.275850259	0.007979195	HSC
Myb	-0.286459327	0.006571747	HSC
Arhgef40	-0.287242399	0.002298689	HSC
Dcaf6	-0.289340415	0.007634069	HSC
Hmgcr	-0.292719326	0.009313342	HSC
Snrnp48	-0.300453904	0.00109096	HSC
Birc3	-0.315231118	0.000592631	HSC
Chd3	-0.31750903	0.002676451	HSC
Cacna1a	-0.320313969	0.00605024	HSC
D11Wsu47e	-0.324547424	0.005679716	HSC
Klf9	-0.325151382	0.003019035	HSC
Ccar1	-0.327814161	0.001452025	HSC
Srfbp1	-0.331000534	0.004637633	HSC
BC005561	-0.33208504	0.004685981	HSC
Hspa4l	-0.337313678	0.008700962	HSC
Susd6	-0.34098912	0.004005932	HSC
Smim5	-0.345489409	0.009914814	HSC
Arhgef28	-0.353147862	0.002751353	HSC
Ryr3	-0.353303963	0.007478435	HSC
Epb41l3	-0.356409874	0.002252984	HSC
Fundc1	-0.357022006	0.005398942	HSC
Ank2	-0.367248339	0.002727862	HSC
Uba5	-0.370758584	0.007158263	HSC
Aopep	-0.374447314	0.005243294	HSC
Arhgap11a	-0.378241906	0.009380889	HSC
Micall2	-0.381125488	0.003430169	HSC
Rmi1	-0.381362054	0.009317533	HSC
Pik3cd	-0.386090415	0.003624117	HSC

Neb	-0.388122695	0.002921278	HSC
Cox17	-0.388140655	0.001220645	HSC
Rpain	-0.390332099	0.001626922	HSC
Podxl	-0.395485564	0.007030731	HSC
Dnah9	-0.395712529	0.006632936	HSC
Cep131	-0.396454348	0.008174303	HSC
Trpm3	-0.400669516	0.00733658	HSC
Pex26	-0.408138803	0.004317216	HSC
Gm37006	-0.413575904	0.009279097	HSC
Zfp182	-0.414533947	0.000632342	HSC
H2-K2	-0.41709111	0.001373509	HSC
Grm8	-0.419507336	0.004020439	HSC
Gm11168	-0.422647874	0.007387131	HSC
Mdga2	-0.426686342	0.008448065	HSC
Snx11	-0.427171747	0.002789146	HSC
Amigo2	-0.430703507	0.002643551	HSC
Veph1	-0.431384452	0.005192251	HSC
Hivep2	-0.43163804	0.002834092	HSC
Fchsd1	-0.432516274	0.002233514	HSC
Col11a2	-0.433884736	0.003224045	HSC
Zfp821	-0.434392376	0.006846689	HSC
Magt1	-0.435697984	0.000190957	HSC
Taf7	-0.437161913	0.001347396	HSC
Tbc1d10a	-0.439839127	0.007964254	HSC
Serpinh1	-0.444550301	0.008507003	HSC
Il15	-0.445859551	0.001229139	HSC
Dnah7c	-0.448267626	0.009769458	HSC
C230004F18Rik	-0.454842702	0.00718331	HSC
St8sia4	-0.461499052	0.004856452	HSC
Mrps31	-0.463236853	0.000803948	HSC
Egflam	-0.465153673	0.007908162	HSC
Eln	-0.46763759	0.005643619	HSC
Ticam2	-0.468567129	0.008238127	HSC
Rassf4	-0.468940632	0.005216058	HSC
Plod1	-0.469002909	0.001092969	HSC
Gm10721	-0.469031184	0.005008726	HSC
Hdx	-0.469625806	0.004644246	HSC
4732440D04Rik	-0.470777188	0.002875547	HSC
Etl4	-0.473467245	0.004195382	HSC
Nfkbia	-0.476158571	0.005077741	HSC
Tro	-0.479216253	0.004997331	HSC
Ttll7	-0.483121401	0.008293965	HSC
Hspa8	-0.484256964	0.001716326	HSC

Nlrp12	-0.485727615	0.00681889	HSC
Csrnp2	-0.488325349	0.004892342	HSC
Lamc2	-0.490877119	0.002959955	HSC
Pfkfb3	-0.491462312	0.003287272	HSC
4930432E11Rik	-0.491757903	0.002012937	HSC
Ogfod2	-0.492195129	0.005528158	HSC
Rabl2	-0.492935686	0.006720133	HSC
Itga11	-0.494573969	0.008342341	HSC
Dnajc28	-0.496269089	0.002275678	HSC
Rian	-0.496585537	0.000667986	HSC
Srek1ip1	-0.497247907	0.005009328	HSC
Ccdc112	-0.497499997	0.000748612	HSC
Papln	-0.497979081	0.006716037	HSC
Cited2	-0.498641245	0.002593551	HSC
Sycp2l	-0.502122557	0.008688488	HSC
Tchp	-0.502349428	0.003610594	HSC
Ltb	-0.509670601	0.001420159	HSC
Vwa3a	-0.509690652	0.004240959	HSC
Pik3ip1	-0.51183915	0.009084121	HSC
Hspa5	-0.512848882	0.000402941	HSC
Zfpm2	-0.51321066	0.004323488	HSC
Dysf	-0.517238952	0.002161147	HSC
Lemd1	-0.51978402	0.008604275	HSC
Abtb2	-0.519910629	0.005127269	HSC
Trim14	-0.520308527	4.48E-08	HSC
Acrbp	-0.521206981	0.008019284	HSC
Fam83g	-0.522481886	0.002078581	HSC
Zdhhc14	-0.524067028	0.00497138	HSC
Paqr3	-0.52441068	0.006775209	HSC
Nsl1	-0.526963327	0.00119479	HSC
Tnni3k	-0.527575138	0.009982145	HSC
Hexim1	-0.52898974	2.76E-05	HSC
Ctcfl	-0.529359922	0.009826905	HSC
Klhl29	-0.529707486	0.007110191	HSC
Morc1	-0.529868313	0.000795314	HSC
Dusp27	-0.529896109	0.005015816	HSC
Sec24d	-0.530966889	0.005593705	HSC
Syt2	-0.531741817	0.001578526	HSC
Gm43323	-0.533162773	0.006701382	HSC
6330549D23Rik	-0.534088715	0.008312786	HSC
Folr1	-0.536564169	0.005635418	HSC
Hist1h4m	-0.537600996	0.007181786	HSC
Gm36640	-0.538554678	0.009245105	HSC

Celf6	-0.540204327	0.00777836	HSC
Sh3bgrl2	-0.540371186	0.006259958	HSC
Cilp	-0.543075676	0.006188048	HSC
Stra6l	-0.545710778	0.004575835	HSC
Cfap52	-0.549880562	0.008023132	HSC
She	-0.555001707	0.003094215	HSC
Gfi1	-0.555018384	0.005432576	HSC
Dpyd	-0.556704384	0.002851058	HSC
Nfil3	-0.558130549	0.005717894	HSC
Vmn2r8	-0.559895749	0.003030324	HSC
Ccdc7a	-0.562509893	0.007733256	HSC
Acvr1c	-0.562688943	0.004769027	HSC
Rnf225	-0.563616639	0.008792823	HSC
Snx7	-0.56520476	0.000390717	HSC
Slamf7	-0.568155457	0.006045834	HSC
B330016D10Rik	-0.569054454	0.002674156	HSC
Slc5a9	-0.569736749	0.003175603	HSC
4933432K03Rik	-0.573481705	0.005114369	HSC
Jph1	-0.576290103	0.00124066	HSC
Gm37474	-0.576615757	0.000204118	HSC
Vmn2r105	-0.577529301	0.004485599	HSC
D730005E14Rik	-0.578009706	0.009053217	HSC
Frmpd2	-0.579823703	0.008577102	HSC
4921509C19Rik	-0.582790251	0.0055285	HSC
Gm5739	-0.586201802	0.005894586	HSC
Phkg1	-0.58680176	0.003447129	HSC
Cacnb4	-0.586846273	0.003257	HSC
Wdr49	-0.586871896	0.006272338	HSC
Gm14410	-0.591276552	0.00251978	HSC
Gm19139	-0.591756642	0.003202694	HSC
Slc12a1	-0.592350874	0.007753903	HSC
Gm12504	-0.592403261	0.007394009	HSC
Tmod1	-0.593150888	0.00237947	HSC
Cdk15	-0.593924645	0.005081316	HSC
Serpina3g	-0.596328836	0.005918318	HSC
Nek5	-0.596340133	0.00247538	HSC
9930024M15Rik	-0.596906095	0.001268898	HSC
Impg1	-0.597072476	0.006656021	HSC
Kcna4	-0.598422349	0.003938591	HSC
Bub1	-0.599482491	0.009946444	HSC
Gm15601	-0.602145165	0.008913764	HSC
Fam155a	-0.603170081	0.006927282	HSC
Gm10767	-0.60398601	0.003200492	HSC

Gm43386	-0.604139647	0.007051076	HSC
Vmn1r171	-0.605554944	0.001402655	HSC
Gm49783	-0.607793038	0.000944204	HSC
Gm15886	-0.6080062	0.004270454	HSC
Gja5	-0.608967904	0.006225462	HSC
Gm19085	-0.611146768	0.006735354	HSC
Bnc2	-0.611703646	0.002111838	HSC
Gale	-0.612371214	0.008321819	HSC
Gm2420	-0.612553625	0.008220915	HSC
Wnt8b	-0.614114278	0.007383498	HSC
Fezf2	-0.614318353	0.006771091	HSC
Haus8	-0.614714382	0.001051015	HSC
Carhsp1	-0.616899557	0.006166096	HSC
Resf1	-0.617000611	0.001077644	HSC
Dnaja1	-0.619098647	0.000589263	HSC
5330413P13Rik	-0.620067647	0.001517469	HSC
Slc6a5	-0.620231724	0.005494224	HSC
Tmem178b	-0.621333722	0.000958804	HSC
Ccdc110	-0.62151357	0.003450455	HSC
Hemgn	-0.623480635	0.001852161	HSC
Vmn2r89	-0.624264102	0.004453477	HSC
Etohd2	-0.624462217	0.004512116	HSC
9330159F19Rik	-0.627427062	0.004737558	HSC
Accsl	-0.629047361	0.003023106	HSC
Noxred1	-0.629994139	0.00966802	HSC
Gm47218	-0.630117033	0.007107799	HSC
Pknox2	-0.632842682	0.009464163	HSC
AC113540.1	-0.632934191	0.006560802	HSC
Gm5535	-0.633156209	0.001717916	HSC
Ccdc85a	-0.633474237	0.008875742	HSC
Eno3	-0.634049964	0.000666864	HSC
AC121957.1	-0.634742371	0.009342957	HSC
Aldh3b3	-0.634838612	0.005396984	HSC
Gm19409	-0.634919668	0.008783215	HSC
Ripor3	-0.634922215	0.00265807	HSC
Gm50012	-0.636169211	0.001225428	HSC
Vmn2r-ps54	-0.639855418	0.003278758	HSC
Wfikkn2	-0.640265069	0.009941992	HSC
Amph	-0.641572122	0.001066184	HSC
Gm30382	-0.642507679	0.009234082	HSC
Gpr158	-0.643099367	0.002852614	HSC
Scn9a	-0.644676777	0.001237867	HSC
Gm39041	-0.645574817	0.003962667	HSC

Cyb561	-0.646820916	0.000935252	HSC
Gm37297	-0.649350739	0.008629576	HSC
Gm38413	-0.653598202	0.007632663	HSC
Gm8369	-0.653783183	0.006055829	HSC
Neurod1	-0.654651181	0.007964831	HSC
8030445P17Rik	-0.655183088	0.004831825	HSC
Cyp2j13	-0.655922878	0.003862268	HSC
Cdca3	-0.658739932	0.006395149	HSC
Gm19829	-0.659988072	0.006665171	HSC
Cmc2	-0.660429592	0.000974139	HSC
Krt14	-0.662860767	0.008334711	HSC
Dnaic1	-0.664627278	0.002696606	HSC
Vmn1r73	-0.664968644	0.002060677	HSC
G6pc	-0.665401577	0.002414771	HSC
Banp	-0.665869464	9.40E-05	HSC
Tulp2	-0.666202606	0.007317304	HSC
Adamts13	-0.666984398	0.009197802	HSC
Arhgap22	-0.667867916	0.000445937	HSC
Gm18367	-0.668019393	0.005507008	HSC
Trim38	-0.668230792	0.008103581	HSC
Gm44597	-0.668523078	0.006015752	HSC
Slc2a7	-0.668996197	0.003959965	HSC
Phxr4	-0.672003826	0.006173838	HSC
Gm10845	-0.674028985	0.005010778	HSC
Spag6l	-0.674198596	0.009353765	HSC
Gm20745	-0.67421019	0.003978231	HSC
Vmn2r-ps159	-0.676686332	0.005915639	HSC
Cabp4	-0.678197164	0.00709363	HSC
Mettl21c	-0.68013878	0.001852992	HSC
Slc17a4	-0.681034332	0.001304486	HSC
Gm4961	-0.68262703	0.004910531	HSC
Krt20	-0.683446683	0.001536532	HSC
Gm38412	-0.68448593	0.002814899	HSC
Fcer2a	-0.692092374	0.000476603	HSC
Slfn4	-0.693370982	0.00101994	HSC
Gm29055	-0.695356176	0.009099662	HSC
D630003M21Rik	-0.695425483	0.000967373	HSC
Espnl	-0.69665748	0.00155227	HSC
Gm45629	-0.697298174	0.003704081	HSC
Vmn1r59	-0.697474661	0.001665313	HSC
Dytn	-0.699449221	0.000601959	HSC
Gm2670	-0.699558502	0.005147934	HSC

Gm35835	-0.699828751	0.000553896	HSC
Gm35823	-0.700754106	0.007232543	HSC
Tsc22d3	-0.701255807	0.001663724	HSC
9930038B18Rik	-0.701272568	0.003377264	HSC
Gm43414	-0.702901176	0.006097472	HSC
Tceal3	-0.703883055	0.000837445	HSC
Cdh16	-0.703942796	0.004705793	HSC
Dynll1	-0.704191234	2.45E-06	HSC
Cfap58	-0.704214282	0.000735273	HSC
Gm37120	-0.704371789	0.00420299	HSC
Scara5	-0.707366096	0.000654836	HSC
Ccne2	-0.708511049	0.005928592	HSC
Kynu	-0.709770383	0.005460392	HSC
Nek2	-0.709910884	0.000113745	HSC
Hsd3b5	-0.710030356	0.003460197	HSC
Gm19094	-0.71367496	0.008044547	HSC
Bpifb6	-0.71493578	0.006970834	HSC
Gm11389	-0.715374803	0.004445037	HSC
Cdc20	-0.717547418	0.000450842	HSC
Bex2	-0.720907492	0.002177018	HSC
Tmprss2	-0.721831585	0.000290225	HSC
Llph-ps1	-0.723279615	0.001151914	HSC
Olfr740	-0.723524413	0.002577296	HSC
Trim30c	-0.723853672	0.000340999	HSC
Rasgef1b	-0.727132261	3.69E-05	HSC
Pim2	-0.728937071	4.41E-05	HSC
Adcy5	-0.728961119	0.001170904	HSC
4933401D09Rik	-0.729817748	0.000851467	HSC
Gm30292	-0.730274998	0.000517855	HSC
Anks4b	-0.731205776	0.000213316	HSC
Gm26762	-0.731710542	0.001232886	HSC
Vmn1r72	-0.738170098	0.006521067	HSC
Ms4a8a	-0.738215495	0.003724998	HSC
Lrtm1	-0.739005139	0.000281436	HSC
Gm36101	-0.740282996	0.007393369	HSC
Gm13425	-0.740934333	0.009812585	HSC
Slc13a2os	-0.742762301	0.000590855	HSC
Cxcr4	-0.745079642	5.05E-05	HSC
Gm12589	-0.745836516	0.002945159	HSC
Rassf10	-0.746677952	0.000983116	HSC
Il4	-0.746764064	0.009728049	HSC
Gm28530	-0.748112253	0.000302598	HSC
Slc6a7	-0.748840277	0.0026421	HSC

Ccdc80	-0.749633874	0.001198703	HSC
Il23a	-0.752168212	0.000586279	HSC
Dnajc24	-0.752438721	3.65E-06	HSC
4930429H19Rik	-0.753096773	0.002479943	HSC
Ddt	-0.753194149	0.009155217	HSC
Arx	-0.756465603	0.002698667	HSC
Olfr90	-0.756551174	0.002621405	HSC
Rtn1	-0.757812428	0.000845861	HSC
Olfr65	-0.757978299	0.000902701	HSC
Rsph4a	-0.759377772	0.000108357	HSC
Tnfrsf11b	-0.760084758	0.00822724	HSC
Gm37310	-0.764164049	0.003548702	HSC
Mvd	-0.765982771	0.006315332	HSC
Hrg	-0.767150068	0.001274928	HSC
Gm1995	-0.76895061	0.006217043	HSC
Mfsd2a	-0.771042624	0.004439267	HSC
Trpc5	-0.773773255	0.001076962	HSC
Gm38056	-0.774811421	0.000218456	HSC
Gm28373	-0.778230251	0.000305746	HSC
Bnipl	-0.781236812	0.000340383	HSC
Gm29114	-0.78315787	0.001450276	HSC
Gm37166	-0.784324116	0.000358249	HSC
Xirp1	-0.786207261	0.000133728	HSC
Opn4	-0.78661773	0.000168772	HSC
Gm33326	-0.801951193	0.000595717	HSC
Gm15538	-0.803032818	0.002901802	HSC
Smtnl1	-0.804382926	0.000565123	HSC
Gm4963	-0.805325846	0.000431933	HSC
Calcr	-0.808048363	0.000536218	HSC
Ctss	-0.810346943	0.00109615	HSC
Spatc1	-0.810486808	0.006307738	HSC
Kcns3	-0.810529672	0.008326262	HSC
Gm15853	-0.810996397	4.76E-05	HSC
Dpysl3	-0.813289235	0.000172929	HSC
Gm6290	-0.81425786	0.002371019	HSC
Gm49689	-0.816782287	0.001610156	HSC
Sgcd	-0.81686539	0.000548969	HSC
Nlrp9b	-0.817255714	0.001420856	HSC
Gdnf	-0.818067881	0.00041753	HSC
Slc16a3	-0.82096095	0.000298236	HSC
Patl2	-0.821533873	0.000661663	HSC
Fgf3	-0.825726961	0.006530247	HSC
1700001L19Rik	-0.828564598	0.003773292	HSC

Mast1	-0.831700642	0.000461294	HSC
4930507D10Rik	-0.831892929	0.008365055	HSC
Gm37664	-0.83792313	0.007219689	HSC
Slc22a12	-0.838763572	0.001616046	HSC
Gdpd5	-0.845285809	0.002136262	HSC
Gm43808	-0.845953173	0.001327148	HSC
Gm38069	-0.850588764	0.000623661	HSC
Gm2673	-0.854407573	0.004468784	HSC
Ntng1	-0.854773205	2.19E-06	HSC
Gm45220	-0.861818427	0.000676163	HSC
Hsph1	-0.862766441	0.000586695	HSC
Pcdh8	-0.863196211	0.002401882	HSC
Far1os	-0.863964987	0.002200395	HSC
2810407A14Rik	-0.865452395	8.77E-05	HSC
Olfr1278	-0.868802391	0.001349169	HSC
Gm13571	-0.871093652	0.000880163	HSC
Gm35974	-0.872912231	0.002479786	HSC
Gm37834	-0.877766028	0.000610773	HSC
Cish	-0.878279558	0.00324403	HSC
Per1	-0.880276371	8.17E-07	HSC
Phactr3	-0.883157909	6.26E-05	HSC
Bhlhe40	-0.883880971	0.009600089	HSC
P2ry1	-0.888909496	0.00251515	HSC
Gm47111	-0.890205023	0.000714309	HSC
Fhl2	-0.890755978	0.000676859	HSC
Krt15	-0.892147542	0.003239759	HSC
1700016C15Rik	-0.89693001	0.002743633	HSC
Cyp2b19	-0.899152768	0.000606178	HSC
Gm16104	-0.899636566	0.000208405	HSC
Gm48475	-0.904312967	0.00011459	HSC
Vmn1r74	-0.906177186	0.00033888	HSC
Olfr267	-0.9095774	0.000335943	HSC
Coro2a	-0.911041013	6.47E-09	HSC
A730020M07Rik	-0.911883727	0.000472822	HSC
Gm7891	-0.917091959	0.000568327	HSC
Gm29776	-0.921197093	0.000130179	HSC
Cyp4a31	-0.92769979	0.000272414	HSC
Gm8258	-0.935240814	0.000363543	HSC
Clic6	-0.937751219	3.26E-05	HSC
Gm2123	-0.938341669	5.64E-05	HSC
Gm33301	-0.941896591	0.000172594	HSC
Cd14	-0.950624266	1.58E-05	HSC
Gm47777	-0.953771712	0.000762121	HSC

Gm39318	-0.956264116	0.003884256	HSC
Olfr1438-ps1	-0.964607867	0.000229246	HSC
Gm20587	-0.975537944	2.46E-05	HSC
Tcf23	-0.982184303	3.08E-05	HSC
C8b	-0.985843125	3.81E-05	HSC
Adamts4	-0.990898511	2.24E-06	HSC
Arhgap20os	-0.991125281	0.00032263	HSC
Gm45894	-0.991330184	2.10E-06	HSC
Loxl4	-1.017538476	0.000234682	HSC
Gm26753	-1.026959225	0.00383965	HSC
Gm8352	-1.053952364	0.001504127	HSC
Nlrp4a	-1.058166839	4.50E-06	HSC
Platr4	-1.064130841	0.000485383	HSC
Azgp1	-1.064413145	6.04E-06	HSC
Gm15666	-1.081322429	1.40E-05	HSC
Gm14239	-1.090439811	1.30E-05	HSC
B230303A05Rik	-1.101377896	9.22E-07	HSC
Gm38368	-1.137245217	1.90E-06	HSC
Xbp1	-1.140770963	1.99E-05	HSC
Sostdc1	-1.14199758	0.000529824	HSC
Gm32122	-1.149419984	9.04E-08	HSC
Socs2	-1.160790009	0.000209509	HSC
Hspa1b	-1.164386018	0.003020899	HSC
Gm24922	-1.297323906	0.006761741	HSC
Il10ra	-1.315071808	1.87E-05	HSC
Slfn2	-1.3437833	0.001258423	HSC
Jchain	-1.498376892	0.00065952	HSC
Igkc	-3.818970416	0.00204516	HSC
Ighm	-5.016847929	7.68E-10	HSC
Gm25834	1.708064182	9.45E-07	CLP
Gm20744	1.681567607	1.01E-06	CLP
1700013F07Rik	1.675573142	0.000195092	CLP
Pah	1.632791162	7.18E-06	CLP
Gm17202	1.584608113	2.08E-05	CLP
Car1	1.582734085	0.00090579	CLP
Gm4166	1.541296402	4.40E-05	CLP
AC102243.1	1.51496071	2.77E-05	CLP
Gm34794	1.512919058	1.38E-06	CLP
Mkrn3	1.472973908	1.84E-05	CLP
Gm17359	1.458530296	0.001368562	CLP
Gm22424	1.448252693	0.001140223	CLP
Gdf3	1.406949291	0.000211756	CLP
Olig3	1.388041277	0.004807986	CLP

Gm5092	1.344157571	1.20E-05	CLP
Atn1	1.340837803	1.26E-05	CLP
AC132253.7	1.332010876	7.55E-05	CLP
mt-Ti	1.325845781	0.008146264	CLP
Gm18878	1.309178007	0.001943771	CLP
A530016L24Rik	1.303937795	0.000591804	CLP
Chil4	1.302279842	9.16E-05	CLP
1110003F10Rik	1.266914116	0.000340034	CLP
Vkorc1	1.258693658	0.00045214	CLP
Gm37800	1.238744088	0.000365677	CLP
Fank1	1.230729492	0.002124273	CLP
Csnka2ip	1.225225168	0.006977089	CLP
Olfr118	1.221439711	0.001048045	CLP
Jchain	1.218152607	0.000106776	CLP
Rab4b	1.187484098	0.000271185	CLP
AC166172.1	1.172475148	0.003776684	CLP
Gm18564	1.170948655	0.006904596	CLP
Gm45051	1.167191893	0.000262885	CLP
D030018L15Rik	1.166404211	0.000890195	CLP
Rdh18-ps	1.165026546	0.003937564	CLP
Prdm8	1.154266267	0.000362562	CLP
Gm45601	1.144748006	0.002084565	CLP
Ache	1.144318868	0.000782024	CLP
Gm9888	1.144027573	0.00488891	CLP
Pramef17	1.143810146	0.004496187	CLP
Fscn3	1.109731792	0.000985231	CLP
Cyp2a5	1.107475385	0.000726624	CLP
Gm37245	1.106909373	0.004324855	CLP
Olfr1398-ps1	1.097028825	0.002511354	CLP
Ccl3	1.089963226	0.004676488	CLP
Gm47322	1.081010734	0.003416928	CLP
Adgre4	1.077739405	0.004914111	CLP
Gm11728	1.070695274	0.001580302	CLP
Itgbl1	1.069847669	0.001718074	CLP
Kcnj4	1.068003927	0.003385177	CLP
Gm11336	1.066340169	0.009877005	CLP
Gm7616	1.058318437	0.002487685	CLP
Zfp804b	1.056200645	0.003424519	CLP
Olfr1295	1.054393616	0.000299181	CLP
Aqp1	1.053476791	0.001433378	CLP
P4ha3	1.046985359	0.006522463	CLP
Lsmem1	1.043465818	0.004016197	CLP
Gm37063	1.042257754	0.000940046	CLP

Gm38307	1.041591288	0.005344185	CLP
Mt1	1.041069981	0.003465562	CLP
E130310I04Rik	1.039036571	0.008348473	CLP
Gm43023	1.030320581	0.004263917	CLP
Rab26os	1.027075954	0.006346623	CLP
Gm8978	1.02582244	0.005272539	CLP
Prdm6	1.02467747	0.002616447	CLP
Gm38021	1.024425281	0.009879478	CLP
Gm47357	1.022148219	0.002669859	CLP
Vmn2r26	1.021402909	0.001595892	CLP
Vmn1r229	1.021197915	0.001069801	CLP
Vmn1r9	1.020899856	0.002220342	CLP
Rhd	1.019269675	0.00953295	CLP
Gm35974	1.015691164	0.002067715	CLP
Inca1	1.01387837	0.006825832	CLP
1500005C15Rik	1.012948387	0.004687398	CLP
Gm43422	1.012895522	0.005428714	CLP
Tmem26	1.009044859	0.00478966	CLP
Ccl2	1.004852261	0.002509897	CLP
Gm44439	1.002928904	0.001872351	CLP
Ap1m2	0.994684073	0.00435125	CLP
Cabp7	0.9916144	0.005118105	CLP
Creb3l1	0.991110556	0.007463348	CLP
Gm35288	0.989965953	0.004489388	CLP
B930036N10Rik	0.985673548	0.001022074	CLP
Gm10465	0.984258658	0.00419533	CLP
Gm49688	0.976562195	0.001650112	CLP
Gm21569	0.973000833	0.00299946	CLP
Gm35842	0.965213222	0.006819558	CLP
Ankrd63	0.961446365	0.002793847	CLP
Btd	0.957627926	0.001911843	CLP
Sox15	0.947701604	0.002971257	CLP
Gm10457	0.947115995	0.001425938	CLP
Gm43465	0.942705571	0.004955912	CLP
C330024D21Rik	0.940667125	0.002819527	CLP
Gm43330	0.940139492	0.008090801	CLP
Gm12708	0.930272596	0.004396025	CLP
Gm29488	0.924031579	0.004125954	CLP
Gm16976	0.920339695	0.002323559	CLP
Gm10658	0.91376771	0.007995261	CLP
Olfr523	0.910338478	0.002566309	CLP
Olfr1396	0.909594907	0.004910311	CLP
Wnt8a	0.908163025	0.003380544	CLP

Mfsd2b	0.900714091	0.001605951	CLP
Zfp990	0.894184339	0.008403192	CLP
Themis	0.884017336	0.001460658	CLP
Tpd52l1	0.879712328	0.007783924	CLP
Mbip	0.870401434	0.004949884	CLP
Sp3os	0.867767221	0.005106622	CLP
Gm815	0.857242196	0.008401374	CLP
Gm49319	0.85296379	0.005799377	CLP
Slc25a34	0.847135431	0.006937815	CLP
Otp	0.845624203	0.009405884	CLP
Olfr1381	0.836297515	0.004443075	CLP
Adam2	0.823096324	0.003033818	CLP
4930590J08Rik	0.813855072	0.0064226	CLP
Gm17300	0.809933471	0.00934607	CLP
Prrg1	0.806878558	0.003480423	CLP
Gm47798	0.801934468	0.004709475	CLP
Ier3ip1	0.793607742	0.00885364	CLP
St8sia6	0.788405715	0.002053758	CLP
Lrig3	0.786214813	0.00825932	CLP
C030013G03Rik	0.769570152	0.007300535	CLP
Kctd14	0.765662084	0.002367411	CLP
Gm13375	0.761163569	0.008495871	CLP
Gm9118	0.755285225	0.008777049	CLP
Bcar1	0.754910073	0.006110817	CLP
Gm49797	0.751516722	0.001946813	CLP
Ak5	0.750459439	0.007012778	CLP
Banf1	0.726906203	0.003528437	CLP
Ppp1r1c	0.712354027	0.006841354	CLP
Nbdy	0.712194274	0.006560793	CLP
Cxxc4	0.712123431	0.002468833	CLP
Tmbim4	0.705576467	0.002333253	CLP
Diras2	0.689039991	0.002745094	CLP
Meak7	0.675464653	0.00529356	CLP
Lrpap1	0.664524447	0.002086135	CLP
Clstn3	0.65737815	0.007802877	CLP
Eny2	0.641650689	0.006553044	CLP
Tmem256	0.631390183	0.006523826	CLP
Ipp	0.607607536	0.002079658	CLP
Rcor2	0.58375869	0.006170856	CLP
Depdc1a	0.547012344	0.006920507	CLP
Hmgb3	0.497616429	0.009077661	CLP
Hnrnpdl	0.484575781	0.006628343	CLP
Zfp512b	0.454889577	0.008698958	CLP

Btg1	-0.491009549	0.002942226	CLP
Mtmr4	-0.518640712	0.0073948	CLP
Zfp53	-0.530810732	0.008058894	CLP
Sdf2l1	-0.582708327	0.003409474	CLP
Disp2	-0.586088032	0.009481727	CLP
Gatb	-0.609999081	0.006790252	CLP
Olfr1307	-0.615915698	0.007764863	CLP
Iqca	-0.641670443	0.005201895	CLP
B3gnt7	-0.661158551	0.008266535	CLP
Hoxb3	-0.668710246	0.006765224	CLP
AC121957.1	-0.676653082	0.009305706	CLP
Atp1a4	-0.680728363	0.007711293	CLP
Hspa8	-0.692659265	0.00376048	CLP
Tmem45a2	-0.705139364	0.005287868	CLP
Scnn1g	-0.708248403	0.009022689	CLP
Duox2	-0.720050021	0.003085686	CLP
1700034P13Rik	-0.730954933	0.008508671	CLP
Rslcan18	-0.732683711	0.006969861	CLP
Tmem101	-0.740542264	0.008172356	CLP
Slc2a6	-0.741525977	0.00765091	CLP
Adgre5	-0.747436869	0.007426367	CLP
Ifit3	-0.752696608	0.003209051	CLP
Traf1	-0.757177919	0.00118142	CLP
Plin2	-0.761191195	0.007735414	CLP
Ctsh	-0.768456405	0.002113876	CLP
Sh3gl2	-0.770345903	0.005601072	CLP
Gm33994	-0.770864416	0.00985533	CLP
2900092N22Rik	-0.785718928	0.003946395	CLP
Eno3	-0.78945156	0.005476904	CLP
Mmp8	-0.796140576	0.008419781	CLP
Gm7224	-0.797147145	0.008232838	CLP
Gm34121	-0.800346014	0.004377721	CLP
Gm19950	-0.800491437	0.006580672	CLP
C2cd6	-0.801746008	0.001715421	CLP
Gm18986	-0.803095699	0.008652808	CLP
Otx1	-0.805069104	0.007407173	CLP
Gm38257	-0.805858319	0.009131041	CLP
Pnma2	-0.810421715	0.003938468	CLP
Olfr390	-0.81352489	0.005539433	CLP
Zfp948	-0.813709338	0.001748713	CLP
Vmn2r27	-0.81522675	0.009283641	CLP
Spata31d1b	-0.8153607	0.001435295	CLP
Gm48678	-0.816405162	0.003064746	CLP

Anp32b-ps1	-0.817371645	0.009290015	CLP
Fhod3	-0.819135833	0.004941552	CLP
Adam8	-0.824195006	0.006100999	CLP
Aif1l	-0.836370728	0.006827127	CLP
Fgf6	-0.837601842	0.007177458	CLP
Gm43343	-0.838090536	0.003006562	CLP
Tinagl1	-0.838457001	0.002621232	CLP
Kctd7	-0.840581037	0.00132431	CLP
Gadl1	-0.841862548	0.005022479	CLP
Zbtb7b	-0.849915946	0.001738037	CLP
Gm29125	-0.851627102	0.007769679	CLP
Gm6866	-0.8567995	0.008083103	CLP
Hoxc10	-0.857077897	0.006411782	CLP
Cpa6	-0.857336302	0.002072477	CLP
AC102790.1	-0.858460357	0.008861719	CLP
Gm40617	-0.863775278	0.009686585	CLP
Wfs1	-0.865205477	0.000386734	CLP
Sec14l2	-0.86793405	0.005597068	CLP
Car14	-0.869588601	0.009823577	CLP
Gm42930	-0.871334289	0.002394907	CLP
Gm37166	-0.871834982	0.00977644	CLP
E130311K13Rik	-0.876614539	0.009401042	CLP
Dusp14	-0.880144575	0.006605649	CLP
Ccr7	-0.880462926	0.000309402	CLP
Syt15	-0.884578197	0.007041741	CLP
1700113H08Rik	-0.88513142	0.001967617	CLP
Serpinb6b	-0.885558744	0.000126335	CLP
Gm4707	-0.885703081	0.008886375	CLP
Tgm7	-0.887806742	0.00373857	CLP
Cpvl	-0.890086089	0.002777387	CLP
Gm48478	-0.892675673	0.004255795	CLP
Arhgef25	-0.89369243	0.000584609	CLP
Kitl	-0.894137821	0.005796861	CLP
4930594C11Rik	-0.894667984	0.007355919	CLP
Ficd	-0.896161573	0.001069749	CLP
Gm5739	-0.899209897	0.003259447	CLP
Lrrn2	-0.90354931	0.001386224	CLP
Gm33472	-0.90356616	0.003338555	CLP
Gm44951	-0.903997708	0.002964126	CLP
Psg18	-0.90561363	0.00362538	CLP
AC158975.2	-0.908708996	0.008336825	CLP
Swsap1	-0.909534274	0.001109337	CLP
Gm13712	-0.911186348	0.001094428	CLP

Gm13446	-0.912615579	0.003764735	CLP
Gm21846	-0.912864633	0.001740762	CLP
Crx	-0.914402436	0.005960323	CLP
A730091E23Rik	-0.916476858	0.009854197	CLP
Gm11732	-0.917700303	0.00570583	CLP
Gm36356	-0.919464042	0.00791869	CLP
Gm37531	-0.921084153	0.000788727	CLP
Krt75	-0.925971028	0.002890484	CLP
Pnp2	-0.927053544	0.003181841	CLP
Rnf222	-0.931094786	0.007943393	CLP
Zc2hc1a	-0.931534572	0.004857081	CLP
Gm45774	-0.932042604	0.009613218	CLP
Myo1a	-0.946173479	0.009572002	CLP
Aldh3b1	-0.952258139	0.002881487	CLP
Gm16726	-0.953184891	0.009111588	CLP
Gm20658	-0.953978097	0.00989444	CLP
Calr4	-0.954683945	0.001715596	CLP
Myoz3	-0.956671776	0.008583104	CLP
Bves	-0.957463549	0.002994964	CLP
Gm15793	-0.959092193	0.00160798	CLP
Gm26902	-0.959735907	0.003474295	CLP
Wnt9a	-0.964228153	0.008126971	CLP
Vstm5	-0.965916383	0.00348745	CLP
Gm13700	-0.966613075	0.006008635	CLP
Gm38259	-0.96849655	0.00538824	CLP
Gm43760	-0.973028842	0.007141108	CLP
Tecrl	-0.97355528	0.009991836	CLP
Tmem114	-0.974607197	0.003442481	CLP
Treml4	-0.975035434	0.003372023	CLP
Gm43860	-0.978670539	0.004283525	CLP
Gm42868	-0.981385142	0.00743029	CLP
Gm41638	-0.984512004	0.009559874	CLP
Smtnl1	-0.986548459	0.009818584	CLP
Gm8674	-0.989400766	0.002316337	CLP
Prss30	-0.99054728	0.009223504	CLP
AC133451.1	-0.993213811	0.007907219	CLP
Gm47836	-0.999870499	0.003832632	CLP
1110019D14Rik	-1.000318577	0.007848641	CLP
Gm12174	-1.000826462	0.001985844	CLP
5330411J11Rik	-1.000843921	0.003893769	CLP
Clcnka	-1.003377657	0.00980343	CLP
Platr4	-1.008158978	0.005725973	CLP
Gm26755	-1.015431919	0.004631402	CLP

Gm9387	-1.017153864	0.005272999	CLP
Gm32736	-1.01835002	0.00926284	CLP
Ceacam18	-1.020488822	0.005042456	CLP
Ramp2	-1.023242631	0.001770279	CLP
AC155261.1	-1.02756923	0.002824235	CLP
Pla2g4d	-1.029336069	0.002655727	CLP
6330403L08Rik	-1.029583395	0.002863851	CLP
Gm11419	-1.03386451	0.001121075	CLP
Pou3f3	-1.034350456	0.002911395	CLP
Gm44415	-1.03733745	0.00457196	CLP
Gm28667	-1.037833672	0.003871501	CLP
AC158924.1	-1.047750438	0.009398313	CLP
Gm48249	-1.052352612	0.000497292	CLP
Rims4	-1.060771356	0.000692229	CLP
Gm14399	-1.066939853	0.000266546	CLP
Prrxl1	-1.067431044	0.000801336	CLP
Gm43065	-1.070376752	0.001708937	CLP
Otub2	-1.073004393	0.007535353	CLP
Tsc22d3	-1.075328567	0.000442485	CLP
Gm34078	-1.079192561	0.000611855	CLP
Gm8588	-1.080678893	0.004030406	CLP
Gm12606	-1.08193932	0.003752669	CLP
6430571L13Rik	-1.083606216	0.003686045	CLP
Ina	-1.097274859	0.000701422	CLP
Gm16234	-1.102410431	0.002602768	CLP
Hsph1	-1.102878202	0.000875394	CLP
Nol3	-1.103681174	1.41E-05	CLP
Hepacam	-1.107609172	0.000421518	CLP
Plpp7	-1.109285746	0.008854617	CLP
Gm42866	-1.122368769	0.004688886	CLP
Angpt4	-1.122379767	0.004433951	CLP
Gm2666	-1.12618603	0.001658856	CLP
AC124716.2	-1.129615232	0.00020991	CLP
Lrrtm3	-1.133480943	0.001519153	CLP
4930571N24Rik	-1.135506196	0.000584962	CLP
Gm14305	-1.13701848	0.003166943	CLP
Mnd1-ps	-1.14023945	0.005115996	CLP
Gm15576	-1.141884005	0.003947325	CLP
Morn3	-1.149864438	0.00373728	CLP
4930429D17Rik	-1.1503903	0.005687998	CLP
Gm8139	-1.162595978	0.002981316	CLP
Gm33533	-1.164444778	0.005189883	CLP
Rpl19-ps7	-1.164943522	0.005222174	CLP

Ereg	-1.16930196	0.001010973	CLP
Gm26742	-1.174393426	0.002779702	CLP
Gm38070	-1.176193213	8.24E-05	CLP
Gm15399	-1.18862353	0.00116374	CLP
AC116759.2	-1.188929982	0.002213516	CLP
Rbm12b1	-1.189592149	1.20E-05	CLP
H2-T-ps	-1.195209613	0.000143954	CLP
Olfr92	-1.198807067	0.00558009	CLP
Gm43522	-1.201168349	0.004179589	CLP
Mettl7b	-1.204065558	0.00475221	CLP
Gm20939	-1.209354607	0.00113175	CLP
Gm15773	-1.215460963	0.007635744	CLP
H2-Bl	-1.215877726	0.000299402	CLP
Barhl1	-1.216182844	9.00E-05	CLP
Gm5091	-1.216301368	0.000978964	CLP
Gm13483	-1.221236917	0.002823877	CLP
Gm14540	-1.225223753	0.009771589	CLP
2810002D19Rik	-1.227787206	0.000175315	CLP
Gm2065	-1.236467014	0.008302085	CLP
Zfp791	-1.238188893	0.00651762	CLP
Gm29999	-1.251165439	0.002041918	CLP
Ddit4	-1.25470374	0.000123065	CLP
Gm4454	-1.265001375	0.000298571	CLP
Uts2	-1.265115018	0.00061639	CLP
Plaur	-1.279786287	0.001380153	CLP
Gm14226	-1.283966984	0.006681526	CLP
Ly6m	-1.294343936	0.00776604	CLP
Ndufaf3	-1.295765291	4.01E-05	CLP
Toporsl	-1.298704159	0.001904154	CLP
2610028E06Rik	-1.302553429	3.23E-05	CLP
C330002G04Rik	-1.308085504	0.000250013	CLP
Ppil6	-1.309096691	0.000459664	CLP
Klra13-ps	-1.313511102	0.008488075	CLP
C79130	-1.33059658	2.92E-05	CLP
Klk13	-1.332255712	0.000116071	CLP
Asprv1	-1.346886191	0.002563968	CLP
Mmp9	-1.348613726	5.87E-06	CLP
Gm48903	-1.353783705	4.50E-05	CLP
9330160F10Rik	-1.357236417	0.000754174	CLP
Ifi30	-1.371301052	9.41E-06	CLP
Gm44257	-1.384800458	0.001295602	CLP
Gm44950	-1.441695065	2.59E-05	CLP
Gm48356	-1.465296627	0.000398166	CLP

Gm43423	-1.469332978	4.48E-05	CLP
Krt17	-1.48031872	5.49E-05	CLP
Apol7c	-1.503835266	0.002726071	CLP
A430108G06Rik	-1.506080501	0.000107761	CLP
Rnase10	-1.511082444	3.65E-06	CLP
Gm38376	-1.521873528	0.000105237	CLP
Gm47641	-1.542256718	0.006691175	CLP
Hyal1	-1.548024245	4.68E-06	CLP
4930526M16Rik	-1.555021079	3.89E-06	CLP
4930449E01Rik	-1.611499276	0.000131612	CLP
Gm30015	-1.636255054	0.000184008	CLP
AI606473	-1.64004151	0.00027315	CLP
Cd83	-1.661054091	4.67E-07	CLP
F2rl2	-1.791971031	1.22E-05	CLP
AC121973.1	-1.910023941	0.000150214	CLP
Gm5320	-2.031403125	0.005169507	CLP
Gm5500	-2.116720625	1.90E-06	CLP
Gm49980	0.916252291	0.002135098	GMP
Lipt2	0.694126591	0.002700639	GMP
Ms4a1	0.678022684	0.00097658	GMP
Kcnt2	0.667684977	0.005270874	GMP
Veph1	0.66186106	0.000852853	GMP
Adam28	0.643387171	0.001426648	GMP
Gxylt2	0.640045125	0.000820767	GMP
Cirbp	0.61226793	0.000832174	GMP
Gm6548	0.608921828	4.30E-05	GMP
Gm26982	0.59526583	0.002640631	GMP
1700019J19Rik	0.587560236	0.00401687	GMP
Zc3h6	0.586731186	9.63E-05	GMP
5930430L01Rik	0.585487855	0.000864428	GMP
Gm49953	0.58367094	0.004914503	GMP
Ninl	0.576013637	0.005174123	GMP
Hsd3b3	0.572794755	0.001793646	GMP
Manbal	0.564778357	0.00151002	GMP
Emp1	0.540573815	0.006893083	GMP
2610203C22Rik	0.54001622	0.009636198	GMP
Ccdc178	0.539131158	0.008293373	GMP
Fbxo36	0.514240601	0.008131104	GMP
Phkg1	0.511594722	0.008060127	GMP
2900076A07Rik	0.486329449	0.005785481	GMP
Ifi47	0.480836337	0.000935629	GMP
Il20rb	0.480730172	0.007931063	GMP
Zfp395	0.48027348	0.00221884	GMP

Eya1	0.474689151	0.003902865	GMP
Zfp984	0.465773813	0.00146841	GMP
Fgd1	0.459375218	0.006059305	GMP
Epm2a	0.455843266	0.009661016	GMP
Eif4a-ps4	0.454273093	0.001966267	GMP
Col4a2	0.439959514	0.009170042	GMP
Myo5c	0.435434445	0.004294881	GMP
Depdc1a	0.407302768	0.006515705	GMP
Dgke	0.401927873	0.007011506	GMP
Arl2bp	0.388667671	0.000722808	GMP
Fancl	0.386745843	0.007041338	GMP
Casc4	0.378727245	0.009989266	GMP
Haus2	0.374726563	0.005631496	GMP
Abcg2	0.366006988	0.002954423	GMP
Slc20a2	0.364295366	0.002295895	GMP
Mrpl36	0.363866346	0.002378068	GMP
Tfb2m	0.354880291	0.003026838	GMP
Pdk1	0.352739109	0.000435819	GMP
Acy1	0.347331856	0.004821855	GMP
Fxn	0.34383028	0.007259481	GMP
Gtpbp10	0.340985937	0.003368425	GMP
Ifih1	0.337611988	0.00286273	GMP
Tmem250-ps	0.332749088	0.00312898	GMP
Steap3	0.326623031	0.009176805	GMP
Atp11c	0.322928455	0.001201436	GMP
Fastkd2	0.320355719	0.006694253	GMP
Nln	0.313966017	0.001102355	GMP
Mettl5	0.312736312	0.006173421	GMP
Taf1b	0.311786952	0.005201179	GMP
Bend3	0.30450839	0.009504079	GMP
Cenpb	0.304503676	0.003873815	GMP
Ddx3x	0.300090427	0.00195094	GMP
Med16	0.297616119	0.004240131	GMP
Tab1	0.296597127	0.00934697	GMP
Hnrnpdl	0.295806727	0.008421272	GMP
Usp14	0.292919255	0.002940616	GMP
Cdca5	0.287074502	0.004496198	GMP
Gpn1	0.285102978	0.005393094	GMP
Zbtb40	-0.290753074	0.004222723	GMP
Malat1	-0.305500218	0.008944132	GMP
Sdccag8	-0.310730987	0.006125395	GMP
Micall1	-0.313160291	0.003477271	GMP
Acbd3	-0.322844389	0.003992868	GMP

Cep131	-0.326862752	0.00971876	GMP
Golga4	-0.338026955	0.005662072	GMP
Ska2	-0.33846142	0.007898541	GMP
Exog	-0.33972532	0.001345267	GMP
Dnajb1	-0.347927593	0.006996257	GMP
Rbm12b2	-0.360294048	0.008316228	GMP
Grik5	-0.365784338	0.008114965	GMP
9430015G10Rik	-0.366772498	0.000232061	GMP
Arid4a	-0.370430502	0.00480611	GMP
Luc7l	-0.37107766	0.001604477	GMP
Bag3	-0.372705095	0.008236724	GMP
Thap2	-0.376814177	0.00283675	GMP
Pkd2	-0.378693877	0.00247773	GMP
Syvn1	-0.390694536	0.000566729	GMP
Nfkb2	-0.393136531	0.000825421	GMP
Il21r	-0.394025381	0.001147609	GMP
Gna15	-0.394760295	0.001763606	GMP
Dnajb9	-0.400540818	0.002782811	GMP
Zfp873	-0.402470551	0.008048289	GMP
Abcc10	-0.404772734	0.006436096	GMP
Cbfa2t3	-0.408988172	0.000309347	GMP
Gm47664	-0.40944121	0.009491959	GMP
Dnaja1	-0.41141835	0.000285634	GMP
Coq10b	-0.423088817	0.008814339	GMP
Ryr1	-0.425517794	0.005374299	GMP
Mycbpap	-0.425683955	0.00800806	GMP
Sirt4	-0.439270602	0.005425303	GMP
Wdcp	-0.4452265	0.000265227	GMP
Ccdc117	-0.451804938	0.002989673	GMP
Vmn1r85	-0.455779001	0.007590221	GMP
Dynll1	-0.459268806	6.69E-06	GMP
BC049715	-0.465259362	0.00788137	GMP
Ttc16	-0.465984635	0.008501482	GMP
Rcn3	-0.466394989	0.00896901	GMP
Hspa8	-0.467351162	0.001357248	GMP
Tmem41b	-0.46744268	0.001076355	GMP
Gm38292	-0.469297199	0.006827709	GMP
Ficd	-0.469604499	0.007218616	GMP
Dennd6b	-0.472331795	0.002469226	GMP
Neil1	-0.47517138	0.007581234	GMP
Itga11	-0.48481381	0.00927577	GMP
Slc4a9	-0.493317315	0.00341603	GMP
Cd200r1	-0.49599277	0.002692997	GMP

Gm44822	-0.498943962		0.000794327	GMP
Gm38357	-0.504676668		0.008476939	GMP
Klrb1c	-0.507902268		0.006118839	GMP
Dcun1d3	-0.508108316		0.000840758	GMP
P2ry1	-0.512168235		0.005174706	GMP
Gm5303	-0.512874773		0.006657148	GMP
Gm48515	-0.522378437		0.004356727	GMP
Ctnna3	-0.524003671		0.00557803	GMP
Cyp4f18	-0.525738703		0.009922139	GMP
Pla2g4c	-0.527049968		0.004347176	GMP
Lig4	-0.531290849		0.006926853	GMP
Iqca1l	-0.539759561		0.009302587	GMP
2310058D17Rik	-0.542752695		0.005896431	GMP
Ptgir	-0.543017841		0.004154734	GMP
Herpud1	-0.543094961		1.10E-06	GMP
Gm38082	-0.543480259		0.004760612	GMP
Myh6	-0.543921718		0.001648033	GMP
Trpm4	-0.551486982		0.004950929	GMP
Siglec1	-0.553028743		0.002592385	GMP
Ttbk1	-0.557293731		0.002120768	GMP
Gm5067	-0.559591002		0.005733699	GMP
2700016F22Rik	-0.560417079		0.009834133	GMP
Gm37589	-0.560422138		0.007586918	GMP
Chkb	-0.560431947		0.005242079	GMP
Mterf1a	-0.5685037		0.00125363	GMP
Gm43466	-0.569449252		0.008236088	GMP
Lfng	-0.569513262		0.001200251	GMP
Selp	-0.571987713		0.003913333	GMP
Gm26660	-0.573563341		0.005354715	GMP
Suox	-0.575109509		0.000588192	GMP
Gm37978	-0.576986591		0.009922807	GMP
Dnajb2	-0.580688788		0.001837777	GMP
Oasl1	-0.580921646		0.002862162	GMP
A130048G24Rik	-0.58978929		0.002203444	GMP
Sh3gl2	-0.60030552		0.004620127	GMP
Fut2	-0.606843411		0.003028347	GMP
Dqx1	-0.608773535		0.001189596	GMP
Gm43668	-0.60963222		0.001216629	GMP
Gm15459	-0.612731764		0.004682573	GMP
Mapk4	-0.617886844		0.005304063	GMP
Alox15	-0.619576694		0.001160582	GMP
Apobr	-0.628111084		2.24E-05	GMP
Pmaip1	-0.62832753		0.000437159	GMP

Gm23734	-0.630138548	0.005083347	GMP
Purg	-0.630752201	0.006405974	GMP
Gm49883	-0.644299398	0.003192703	GMP
Gm8185	-0.649301607	0.00269335	GMP
Gm42895	-0.649926255	0.003425538	GMP
Atg9b	-0.651069338	0.001455295	GMP
Arhgap22	-0.651542055	0.00134457	GMP
Gm42463	-0.654745854	0.001442248	GMP
Rdh12	-0.6549552	0.000800467	GMP
Gm45809	-0.65967609	0.006220036	GMP
Dbn1	-0.663800496	0.001650606	GMP
Caskin2	-0.666247399	7.84E-06	GMP
Acrbp	-0.669065056	0.007367477	GMP
Banp	-0.671855709	8.78E-07	GMP
Hebp1	-0.676104117	0.003858651	GMP
Cdr2	-0.676551792	0.00015199	GMP
Ddit3	-0.686730739	0.001998863	GMP
Gm24601	-0.704528942	0.005817344	GMP
Gm48611	-0.712010363	0.005392929	GMP
Oplah	-0.737403494	0.000139292	GMP
Hsph1	-0.752917393	0.000719207	GMP
Gm15344	-0.780794201	9.67E-05	GMP
Gm9993	-0.788087839	0.000553839	GMP
Dse	-0.798622731	0.000738664	GMP
Gm48706	-0.816024097	0.000816965	GMP
Gm26387	-0.822475172	0.000298806	GMP
Cracr2b	-0.911368065	0.000917072	GMP
Id2	-0.911449968	2.17E-05	GMP
Gm45041	-0.956720251	3.65E-06	GMP
Sostdc1	-1.075564213	1.55E-07	GMP
Hspa1b	-2.688544413	6.00E-09	GMP

Supplementary Table 2: Immunophenotype of populations assayed

Cell Type	Immunophenotype
LSK	$Lin^-Sca1^+cKit^+$
HSC	$Lin^-Sca1^+cKit^+CD34^-Flk2^-$
$SlamF1^{High}$	$Lin^-Sca1^+cKit^+CD34^-Flk2^-CD150^{High}$
$SlamF1^{Low}$	$Lin^-Sca1^+cKit^+CD34^-Flk2^-CD150^{Low}$
$SlamF1^{Neg}$	$Lin^-Sca1^+cKit^+CD34^-Flk2^-CD150^{Neg}$
$MPPFlk2^-$	$Lin^-Sca1^+cKit^+CD34^+Flk2^-$
$MPPFlk2^+$	$Lin^-Sca1^+cKit^+CD34^+Flk2^+$
GMP	$Lin^-Sca1^-cKit^+CD34^+Fcgra^+$
CMP	$Lin^-Sca1^-cKit^+CD34^+Fcgra^-$
MEP	$Lin^-Sca1^-cKit^+CD34^-Fcgra^-$
CLP	Lin^-Il7ra^+ $Flk2^+$
B-cell	Ter119- B220+Mac1-CD3-
T-cell	$Ter119^-$ $B220^-Mac1^-CD3^+$
Neutrophil	$Ter119^-$ $B220^-Mac1^+CD3^-Gr1^{High}SSC\text{-}A^{mid}$
Classical Monocyte/ Inflammatory	$Ter119^-$ $B220^-Mac1^+CD3^-Gr1^{Mid}SSC\text{-}A^{low}$
Non-classical Monocyte	$Ter119^-$ $B220^-Mac1^+CD3^-Gr1^-$ $SSC\text{-}A^{low}$
Eosinophil	$Ter119^-$ $B220^-Mac1^+CD3^-Gr1^{Low\text{-}Mid}SSC\text{-}A^{high}$
