# Supplementary material for: NAD+ augmentation with nicotinamide riboside improves lymphoid potential of Atm−/− and old mice HSCs
Source: NPJ Aging Mech Dis. 2021 Sep 21;7:25. doi: 10.1038/s41514-021-00078-3 (PMC8455618; doi:10.1038/s41514-021-00078-3)
Supplement: Supplementary file 2 — Reporting Summary [file 41514_2021_78_MOESM2_ESM.pdf]

nature portfolio

Corresponding author(s): Beerman, Isabel

Last updated by author(s): 2021_Aug_5

Reporting Summary

Nature Portfolio wishes to improve the reproducibility of the work that we publish. This form provides structure for consistency and transparency in reporting. For further information on Nature Portfolio policies, see our Editorial Policies and the Editorial Policy Checklist.

Statistics

For all statistical analyses, confirm that the following items are present in the figure legend, table legend, main text, or Methods section.

n/a	Confirmed	
☐	☒	The exact sample size (*n*) for each experimental group/condition, given as a discrete number and unit of measurement
☐	☒	A statement on whether measurements were taken from distinct samples or whether the same sample was measured repeatedly
☐	☒	The statistical test(s) used AND whether they are one- or two-sided<br>*Only common tests should be described solely by name; describe more complex techniques in the Methods section.*
☐	☒	A description of all covariates tested
☐	☒	A description of any assumptions or corrections, such as tests of normality and adjustment for multiple comparisons
☐	☒	A full description of the statistical parameters including central tendency (e.g. means) or other basic estimates (e.g. regression coefficient) AND variation (e.g. standard deviation) or associated estimates of uncertainty (e.g. confidence intervals)
☐	☒	For null hypothesis testing, the test statistic (e.g. *F*, *t*, *r*) with confidence intervals, effect sizes, degrees of freedom and *P* value noted<br>*Give P values as exact values whenever suitable.*
☒	☐	For Bayesian analysis, information on the choice of priors and Markov chain Monte Carlo settings
☒	☐	For hierarchical and complex designs, identification of the appropriate level for tests and full reporting of outcomes
☒	☐	Estimates of effect sizes (e.g. Cohen's *d*, Pearson's *r*), indicating how they were calculated

Our web collection on statistics for biologists contains articles on many of the points above.

Software and code

Policy information about availability of computer code

Data collection	FACS data was collected on BD FACS Aria II, Fusion or Canto II using Diva software. RNA-seq libraries were sequenced on illunima HiSeq 2500 using 2 × 105 bp reads.
Data analysis	FACS Analysis was performed in FloJo v10 (TreeStar) and statistics data was generated in Prism8. RNA seq data analysis was done by open source tools including STAR, edgeR, and RUV (https://github.com/genomicspark/RNA-seq_QC_analysis).

For manuscripts utilizing custom algorithms or software that are central to the research but not yet described in published literature, software must be made available to editors and reviewers. We strongly encourage code deposition in a community repository (e.g. GitHub). See the Nature Portfolio guidelines for submitting code & software for further information.

Data

Policy information about availability of data

All manuscripts must include a data availability statement. This statement should provide the following information, where applicable:

- Accession codes, unique identifiers, or web links for publicly available datasets
- A description of any restrictions on data availability
- For clinical datasets or third party data, please ensure that the statement adheres to our policy

RNA sequencing data is available on GEO with accession number GSE147662.

Field-specific reporting

Please select the one below that is the best fit for your research. If you are not sure, read the appropriate sections before making your selection.

☒ Life sciences ☐ Behavioural & social sciences ☐ Ecological, evolutionary & environmental sciences

For a reference copy of the document with all sections, see nature.com/documents/nr-reporting-summary-flat.pdf

Life sciences study design

All studies must disclose on these points even when the disclosure is negative.

Sample size	We used power calculations from previous experiments to determine the sample size.
Data exclusions	No data were excluded from the analyses.
Replication	For RNA-seq, there are at least 3 repeats for each group, and PCA analysis confirms strong reproducibility.
Randomization	The mice used in this study were randomly allocated to different experiment group.
Blinding	The investigators were blinded to group allocation during data analysis.

Reporting for specific materials, systems and methods

We require information from authors about some types of materials, experimental systems and methods used in many studies. Here, indicate whether each material, system or method listed is relevant to your study. If you are not sure if a list item applies to your research, read the appropriate section before selecting a response.

Materials & experimental systems

n/a	Involved in the study	
☐	☒	Antibodies
☒	☐	Eukaryotic cell lines
☒	☐	Palaeontology and archaeology
☐	☒	Animals and other organisms
☒	☐	Human research participants
☒	☐	Clinical data
☒	☐	Dual use research of concern

Methods

n/a	Involved in the study	
☒	☐	ChIP-seq
☐	☒	Flow cytometry
☒	☐	MRI-based neuroimaging

Antibodies

Antibodies used	All the information for antibodies used in this study was included in methods part.
Validation	All the antibodies used in this study have ether a validation statement or relevant citation according to manufacture's website.

Animals and other organisms

Policy information about studies involving animals; ARRIVE guidelines recommended for reporting animal research

Laboratory animals	ATM mice used in this study were from a cross of het strain (B6;129S4-Atmtm1bal/J). A minimum of 4, max of 6 littermates homozygous and WT animals were used in the treated and untreated conditions. Both male and female animals were used in all conditions of ATM transplants due to low numbers. but all groups included both sexes. Animals were 2-3 months of age at the time of experiment.<br>C57BL/6 mice are all males. Young mice were 3-4 months old and old mice were 24-29 months old.<br>B6.SJL-Ptprca Pepcb/BoyJ (CD45.1) recipient mice are all females, and 10-12 weeks of age at time of irradiation and transplant.
Wild animals	This study did not involve wild animals.
Field-collected samples	This study did not involve samples collected from the field.
Ethics oversight	Institutional Animal Care and Use Committees (National Institute on Aging)

Note that full information on the approval of the study protocol must also be provided in the manuscript.

Flow Cytometry

Plots

Confirm that:

☒ The axis labels state the marker and fluorochrome used (e.g. CD4-FITC).

☒ The axis scales are clearly visible. Include numbers along axes only for bottom left plot of group (a 'group' is an analysis of identical markers).

☒ All plots are contour plots with outliers or pseudocolor plots.

☒ A numerical value for number of cells or percentage (with statistics) is provided.

Methodology

Sample preparation	For peripheral blood sample preparation, blood samples were treated with ACK twice to remove red blood cells, then stained with antibody cocktail.<br>For whole bone marrow sample preparation, bones from legs and/or arms were crushed and filtered. Then bone marrow cells were treated with ACK once to remove red blood cells then stained with antibody cocktail.<br>For c-kit enrichment and cell sorting, bone marrow cells were positive selected using PE-c-kit antibody and EasySep™ PE Positive Selection Kit II, then stained with antibody cocktail.
Instrument	BD FACS Aria II, BD FACS Fusion or BD FACS Canto II
Software	FACS data was collected on BD FACS Aria II, Fusion or Canto II using Diva software. FACS Analysis was performed in FloJo v10 (TreeStar) and statistics data was generated in Prism8.
Cell population abundance	After sorting, sorted cells were reanalyzed, and the frequency of relevant cells is above 99% (number of relevant cells divided by number of live cells)
Gating strategy	Included in Supplementary Figure 1 and 3.

☒ Tick this box to confirm that a figure exemplifying the gating strategy is provided in the Supplementary Information.
